# Dynamics of the chili pepper transcriptome during fruit development

**DOI:** 10.1186/1471-2164-15-143

**Published:** 2014-02-21

**Authors:** Luis A Martínez-López, Neftalí Ochoa-Alejo, Octavio Martínez

**Affiliations:** 1 Laboratorio Nacional de Genómica para la Biodiversidad (LANGEBIO), Centro de Investigación y de Estudios Avanzados del Instituto Politécnico Nacional (Cinvestav), 36821 Irapuato, Guanajuato, México; 2 Departamento de Ingeniería Genética de Plantas, Unidad de Biotecnología e Ingeniería Genética, Centro de Investigacíon y de Estudios Avanzados del Instituto Politécnico Nacional (Cinvestav-Unidad Irapuato), 36821 Irapuato, Guanajuato, México; 3 Departamento de Biotecnología y Bioquímica, Unidad de Biotecnología e Ingeniería Genética, Centro de Investigacíon y de Estudios Avanzados del Instituto Politécnico Nacional (Cinvestav-Unidad Irapuato), 36821 Irapuato, Guanajuato, México

**Keywords:** *Capsicum*, Chili pepper, Transcriptome, Fruit development, Fruit ripening, Gene expression, RNA-Seq, High-throughput sequencing, Pathway analysis, Illumina MiSeq

## Abstract

**Background:**

The set of all mRNA molecules present in a cell constitute the transcriptome. The transcriptome varies depending on cell type as well as in response to internal and external stimuli during development. Here we present a study of the changes that occur in the transcriptome of chili pepper fruit during development and ripening.

**Results:**

RNA-Seq was used to obtain transcriptomes of whole Serrano-type chili pepper fruits (*Capsicum annuum* L.; ‘Tampiqueño 74’) collected at 10, 20, 40 and 60 days after anthesis (DAA). 15,550,468 Illumina MiSeq reads were assembled *de novo* into 34,066 chili genes. We classified the expression patterns of individual genes as well as genes grouped into Biological Process ontologies and Metabolic Pathway categories using statistical criteria. For the analyses of gene groups we added the weighted expression of individual genes. This method was effective in interpreting general patterns of expression changes and increased the statistical power of the analyses. We also estimated the variation in diversity and specialization of the transcriptome during chili pepper development. Approximately 17% of genes exhibited a significant change of expression in at least one of the intervals sampled. In contrast, significant differences in approximately 63% of the Biological Processes and 80% of the Metabolic Pathways studied were detected in at least one interval. Confirming previous reports, genes related to capsaicinoid and ascorbic acid biosynthesis were significantly upregulated at 20 DAA while those related to carotenoid biosynthesis were highly expressed in the last period of fruit maturation (40–60 DAA). Our RNA-Seq data was validated by examining the expression of nine genes involved in carotenoid biosynthesis by qRT-PCR.

**Conclusions:**

In general, more profound changes in the chili fruit transcriptome were observed in the intervals between 10 to 20 and 40 to 60 DAA. The last interval, between 40 to 60 DAA, included 49% of all significant changes detected, and was characterized predominantly by a global decrease in gene expression. This period signals the end of maturation and the beginning of senescence of chili pepper fruit. The transcriptome at 60 DAA was the most specialized and least diverse of the four states sampled.

## Background

The set of all RNA molecules transcribed in an organ or tissue at a particular point of time under a given set of environmental conditions constitute the transcriptome. In contrast to the genome, which remains largely constant during the life of an individual, the transcriptome is highly dynamic. Global patterns of gene expression vary greatly in space (within different cell or tissue types) as well as in time (during development or due to changing environmental conditions). The transcriptome is characterized by both qualitative features, such as a description of which genes are transcribed, as well as quantitative features including the level of expression of each gene. Evaluating the transcriptome, i.e. estimating the level of expression of all genes under particular conditions, is a key step to understand the complex processes that occur during development.

RNA-Seq [1] is a robust technology to obtain genome-wide estimates of relative gene expression. RNA is purified from a sample of interest and converted to a cDNA library which is sequenced using one of a variety of high-throughput sequencing methods. Sequence fragments are then mapped to a reference genome and the frequency of these alignments is used to estimate the expression of each gene [2]. When a published reference genome is not available, the cDNA reads can be assembled *de novo* in order to obtain a high quality reference to which the reads can be re-mapped [3]. RNA-Seq experiments demand a careful design including replicates that permit the estimation of statistical error or variation that is not explained by the experimental treatment [4].

One of the challenges of high-throughput experiments is the analysis of very large datasets in order to extract biologically pertinent knowledge [5]. Conclusions drawn from the expression of a single gene or a small set of related genes may lead to an incomplete understanding of particular phenomena. A current trend in developmental biology research is to interpret changes in gene expression in the context of simultaneous changes in the resulting proteome and the set of metabolites present during each state of development [6]. As mentioned in [7], “simply comparing genes to themselves have the pitfall of taking molecular information out of context. Numerous scientists have emphasized the need for better context. This can be achieved through holistic measurements of differential connectivity in addition to, or in replacement, of differential expression”. Here we propose to achieve a better understanding of the dynamic changes of the transcriptome by examining the weighted expression of groups of genes related with specific biological processes or metabolic pathways. In addition, we examine the changes in the diversity and specialization of the transcriptome. These analyses create an interpretation framework for the results, which makes it easier to appreciate their meaning.

Chili pepper (*Capsicum* spp.) is one of the most important horticultural vegetable crops worldwide as well as a good model for the study of secondary metabolism during fruit development. *Capsicum* species (approximately 30) are members of the Solanaceae family, which also includes other important crops such as tomato (*Solanum lycopersicum*), potato (*Solanum tuberosum*), tobacco (*Nicotiana tabacum*), eggplant (*Solanum melongena*), and ornamentals like petunia (*Petunia* spp.). Chili pepper fruits synthesize and accumulate a number of valuable compounds including capsaicinoids (responsible for the characteristic “heat” of chilis), pigments such as anthocyanins and carotenoids as well as vitamins A, B and C [8-10]. Because of the agricultural importance of chili peppers, efforts have been made to study the transcriptome of *Capsicum* species as a source of basic and applied knowledge. For example, a chili pepper (*Capsicum annuum* L., cv. Bukang) EST database built from 122,582 sequenced ESTs and 116,412 refined ESTs from 21 cDNA libraries representing 11 different tissues, developmental stages or plants subjected to different stress conditions has been reported [11] and specific efforts have been made to identify genes involved in the biosynthesis of capsaicinoids [12,13]. A transcriptome profile of red chili pepper fruits (*Capsicum annuum* L., TF68) was obtained using 454 GS-FLX pyrosequencing and 33,530 total unigenes were assembled. In this assembly, 30% of aligned reads were assigned to a locus with a specific function annotation, 24% of alignments matched to genes of unknown or unclassified function and 46% could not be aligned to an individual gene. Furthermore, 1,536 single nucleotide polymorphisms (SNPs) and 758 simple sequence repeats (SSRs) were detected that will be useful as molecular markers for linkage mapping and association mapping [14]. Assemblies of two chili pepper transcriptomes from sequences generated by Sanger sequencing (>125,000 ESTs) or the Illumina NGS platform (200 million reads) were carried out to identify SNPs and SSRs as molecular markers useful for breeding or single position polymorphisms (SPPs) for genotyping [15]. Recently, we described a *Capsicum* transcriptome database generated from a hybrid assembly of a collection of ESTs derived from five *Capsicum annuum* organs (root, stem, leaf, flower and fruit) sequenced by the Sanger method and from multiple leaf transcriptomes obtained by pyrosequencing. This project yielded almost 60,000 singletons and 32,314 high quality contigs (75.5% contigs with significant sequence similarity to entries in nucleic acid and protein databases, and 23% not previously described for *C. annuum*) [16]. Transcriptomic studies of chili pepper plants subjected to different stresses have also been reported. For example, 8,525 ESTs were generated and a cDNA microarray analysis identified 613 chili pepper genes responsive to the non-host soybean pustule pathogen *Xanthomonas axonopodis* pv. *glycines*[17]. cDNA microarrays were used to study the expression of ozone stress-regulated genes in a sensitive (*Capsicum annuum* cv. Dabotop) and a resistant chili pepper cultivar (*C. annuum* cv. Buchon) [18]. Transcriptomic analysis of leaves following infection of chili pepper plants with a geminivirus revealed a total of 309 differentially expressed genes between healthy (non-infected) and symptomatic or recovered tissues [19]. More recently, a global transcriptomic analysis of chili pepper plants treated with different biotic/abiotic stresses was carried out to investigate the participation of signaling components (regulons) in both types of stresses. This study targeted the involvement of salicylic acid in the activation of abiotic stress-responsive genes, methyl jasmonate and ethylene in regulating biotic stress-responsive genes, and abscisic acid in regulating both biotic and abiotic stress-responsive genes [20].

Here we present an analysis of the changes in the transcriptome during the development and ripening of chili pepper fruit. We quantified the expression of 34,066 chili genes at each of four time points sampled. Considering these transcriptomes at a more global level, we also evaluated their diversity and specialization, as well as the specificity of the genes expressed during chili fruit development. In addition, by categorizing genes into Biological Process ontologies as well as KEGG metabolic pathways in which they participate, we analyzed the behavior of groups of genes involved in these categories. Finally, our RNA-Seq expression data was validated by analyzing the expression of genes involved in carotenoid biosynthesis by qRT-PCR.

## Results and discussion

### The transcriptome of chili pepper fruit

**Figure 1:**
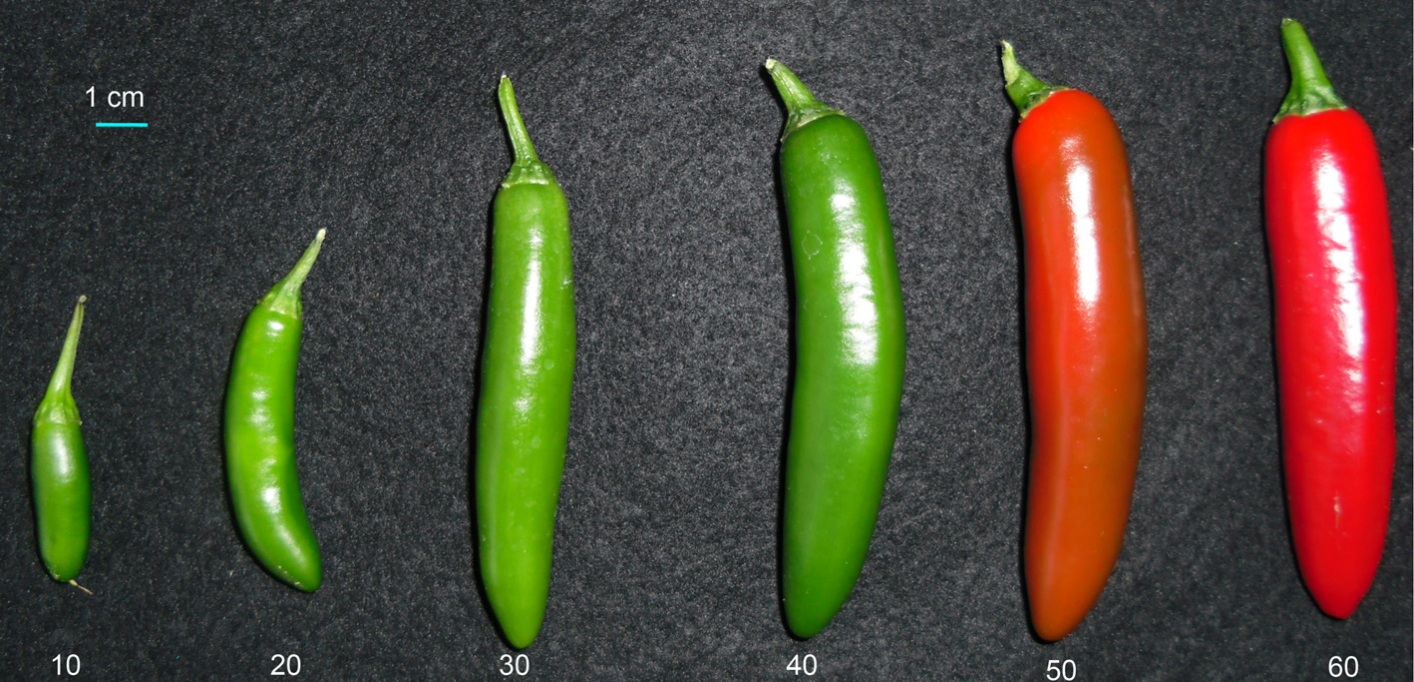
**Chili pepper fruits at different stages of development and maturation.** Representative photographs of chili pepper fruits at 10, 20, 30, 40, 50 and 60 days after anthesis (DAA). Transcriptomes were obtained from the fruits at 10, 20, 40 and 60 DAA.

The transcriptional dynamics of a Serrano type chili pepper cultivar (*Capsicum annuum* L.; ‘Tampiqueño 74’) were studied at four stages of fruit development: 10, 20, 40 and 60 days after anthesis (DAA). Representative photographs of chili pepper fruits at different stages of development, including the time points selected for this work, are presented in Figure 1. These particular developmental stages were selected because 10 and 20 DAA are early and intermediate stages of fruit development, respectively. At 40 DAA, chili fruits reach their maximum size and represent the breaking stage of ripening (mature-green). Finally, 60 DAA is the point at which the fruits are fully ripe [21]. At each time point, mRNA was purified and cDNA libraries created from two independent samplings of whole fruits. These eight libraries were sequenced using the Illumina MiSeq platform, yielding a total of 16,870,295 raw reads, that after quality filtering resulted into 15,550,468 reads of 150 bp in length, a total of 2,333 Mb of data. Reads were trimmed of adaptor sequences and ambiguous bases, then assembled *de novo* into a transcriptome reference using Trinity software [22]. This assembly consisted of 45,505 contigs with a mean length of 1,233 bp. The cDNA reads were re-mapped to the Trinity contigs using Bowtie software [23] and 42,401 contigs had at least one read aligned, indicating that this subset of contigs in the *de novo* assembly represents genes expressed in our chili pepper samples. The contigs were subjected to BLAST analysis against different databases. Contigs with identical BLAST hit were considered as variants (alleles or close paralogs) of the same gene and thus grouped into a single unit for analyses. We obtained evidence for the expression of 34,066 chili genes and 52% of these genes had enough similarity to sequences in databases to provide some annotation (see Tables AF1-1, AF1-2 and AF1-3 in Additional file 1 and Methods Section for details).

#### Estimation of the number of expressed genes and scaled number of mRNA molecules

One difficulty in RNA-Seq transcriptome research is that certain genes can be expressed at such low levels that the total number of cDNA sequences obtained (sequencing depth) can be insufficient to detect them [24]. This problem is similar to one well-known in ecology research when sampling species at a given site; sampling can miss some rare species depending on the sample size [25]. Nonetheless, from the frequencies of expression found in a given sample, it is possible to estimate the total number of genes that are actually expressed in a transcriptome, even if their expression was not detected in the particular sample. We used two such estimators, one originally proposed by Chao *et al.* for ecological research [26] and another proposed by Changjiang *et al.* in the context of gene estimation [27]. An additional parameter of interest is the scaled number of mRNA molecules present in the samples, denoted here by *M* and called “RNA production” in [28]. Comparing the number of reads obtained in a library with the estimated value of *M*, shows how close was the sampling deepness to the scaled number of mRNA molecules existent under each stage of development. To calculate the scaled number of mRNA molecules we used the estimator for *M* proposed by Good [25] in the ecological context. Table 1 presents the total number of sequences obtained in the sampling of the transcriptomes as well as estimations for the number of genes expressed in each transcriptome and the scaled number of mRNA molecules present in each sample, $\hat{M}$.

**Table 1:** **Number of reads, estimated number of genes and scaled number of mRNA molecules (**$\hat{M}$**) per sampling point**

**DAA**	**Reads**	**Estimated number of genes**			$\hat{M}$
		**In sample**	**Chao**	**Changjiang**	
10	1,864,796	24,358	30,451	36,544	1,870,376
20	2,065,216	26,346	32,181	38,016	2,071,181
40	1,781,071	25,533	31,581	37,629	1,786,961
60	2,306,600	23,987	29,433	34,878	2,312,002
Mean	2,004,421	25,056	30,912	36,767	2,010,130
S	234,086	1,083	1,219	1,405	233,944

The number of cDNA reads obtained in our sampling of chili transcriptomes at each stage of development was approximately two million for each sampling point (10, 20, 40 and 60 DAA), with a minimum of approximately 1.8 million and a maximum of approximately 2.3 million (Table 1). These figures are the sum of the number of reads derived from the two biological replicates at each stage. Table 1 also indicates the number of genes showing evidence of expression at each stage of development.

The number of genes for which we obtained evidence of expression at each stage of development ranged from a minimum of 23,987 genes at 60 DAA to 26,346 genes at 20 DAA. The total number of genes whose expression was detected in at least one of the samples was 34,066. This figure likely overestimates the true number of expressed genes because our *de novo* transcriptome assembly strategy results in isoforms or alleles of transcripts expressed from the same locus that are assembled into distinct contigs. Derived estimates of the true number of genes expressed in each one of the stages of development using Chao’s estimator (column “Chao” in Table 1) had a mean of 30,912 with standard deviation of 1,219. From this we concluded that our sampling depth was insufficient to detect the expression of approximately 6,000 genes, which likely have frequencies of expression lower than 0.5 transcripts per million (TPM), and thus were not detected in our data. Estimates of the number of expressed genes obtained with the estimator proposed by Changjiang *et al.* resulted in a larger number of genes whose expression was not detected (around 11,700). It is not clear which of the two estimators is more accurate, but our own simulation studies suggest that the Changjiang *et al.* estimator tends to overestimate the number of undetected genes (unpublished results). The true number of expressed genes is therefore likely to be closer to the number obtained by using Chao’s estimator. Considering that the Arabidopsis genome encodes approximately 27,000 protein coding genes [29], and tomato and potato (Solanaceae) have 30,855 and 32,988 protein-coding genes supported by RNA sequencing respectively [30], we can infer that in this study we detected a large proportion of the genes expressed in the chili fruit transcriptome. The last column of Table 1, $\hat{M}$, indicates estimates of the scaled number of mRNA molecules present at each stage of development, obtained by using Good’s estimator [25]. The estimated values of $\hat{M}$ are very close to the number of reads obtained in the corresponding stages of development (column “Reads”). In all cases the number of cDNA reads obtained represents more than 99% of the estimated $\hat{M}$, indicating that the sample sizes employed in this study are close to the scaled number of mRNA molecules at each stage of development. Therefore, only genes transcribed at very low frequencies, likely less than 0.5 TPM, were possibly missed by the sampling.

#### Genes detected per subset

**Figure 2:**
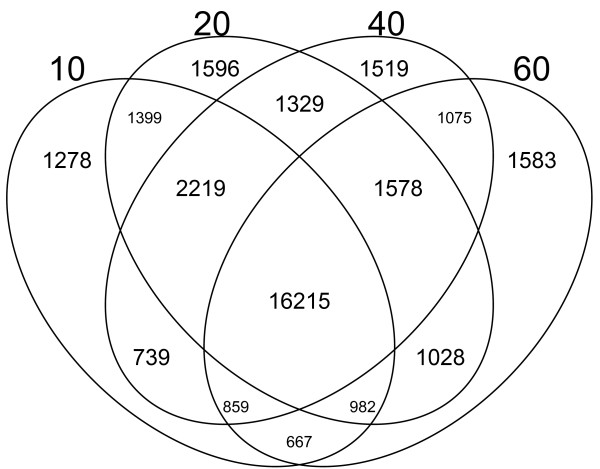
**Venn diagram for the number of genes expressed at each sampling point (10, 20, 40 and 60 DAA).** Each set comprises a sampled stage of development. Numbers in each intersection represent the number of genes detected with at least one read (gene tag) in these disjoint sets (intersections).

Venn diagrams showing the intersections between expressed genes detected at each developmental stage (10, 20, 40 and 60 DAA) are shown in Figure 2. 16,215 genes, or 47.6% of the total of 34,066 genes detected, were expressed in all four stages of development. 5,976 (17.5%) genes were expressed exclusively at a single stage of development. Of these uniquely expressed genes, 1,278 (3.8%), 1,596 (4.7%), 1,519 (4.5%) and 1,583 (4.6%) genes were expressed exclusively at 10, 20, 40 and 60 DAA, respectively. The ratio of genes expressed exclusively at a single stage to that of genes expressed in all stages, 5,976/16,215 or 0.37, indicates that significant changes in expression happen during the development of chili pepper fruit, during which a large proportion of genes are switched on or off. The number of genes whose expression was detected at exactly two stages decreased as a function of time. For example, the number of genes whose expression was detected only during the 10 and 20 DAA stages was 1,399 (4.1%), while the number detected only at 10 and 40 DAA was 739 (2.2%). 667 (2.0%) of genes were expressed only at 10 and 60 DAA.

### Changes in gene expression during fruit development and ripening

Our chili pepper fruit transcriptomes were obtained at consecutive developmental time points (10, 20, 40 and 60 DAA). In our study, we measured changes in gene expression between consecutive time points, specifically between 10 to 20, 20 to 40 and 40 to 60 DAA. We call these changes in patterns of gene expression “transitions”. Change in gene expression in a transition are evaluated by the change in the number of reads detected for a given gene in the corresponding time points. In each transition we determined whether the expression level of each chili gene either decreased (*D*), remained steady (*S*) or increased (*I*). Considering these three possibilities, expression of each gene was classified into 3×3×3=27 patterns, 1−*DDD*,2−*DDS*,⋯,27−*III*, where the order of the letters corresponds to the transition. For example, pattern 8−*DIS* indicates a decrease in expression between 10 and 20 DAA followed by an increase in the interval between 20 and 40 DAA and an steady state between 40 and 60 DAA. To classify each gene into one of the 27 possible patterns we used the R statistical software environment [31] to implement the exact test for differences between two groups of negative-binomial counts proposed in [32] and in [28]. The resulting probabilities were further processed using the Q value procedure [33] at a false discovery rate of 1% (see Methods for details). Details pertaining to the number of genes that were categorized into each one of the 27 possible patterns are presented in Table 2.

**Table 2:** Patterns of expression for genes, biological processes (BP) and metabolic pathways (MP)

**Id**	**Pattern**	**Genes**		**BP**		**MP**	
		**Number**	**%**	**Number**	**%**	**Number**	**%**
1	*DDD*	51	0.15	1	0.11	2	1.32
2	*DDS*	77	0.23	5	0.57	2	1.32
3	*DDI*	11	0.03	6	0.69	4	2.63
4	*DSD*	286	0.84	22	2.51	11	7.24
5	*DSS*	537	1.58	31	3.54	4	2.63
6	*DSI*	145	0.43	43	4.91	3	1.97
7	*DID*	60	0.18	12	1.37	7	4.61
8	*DIS*	64	0.19	5	0.57	2	1.32
9	*DII*	88	0.26	18	2.06	1	0.66
10	*SDD*	146	0.43	17	1.94	3	1.97
11	*SDS*	224	0.66	13	1.49	0	0.00
12	*SDI*	37	0.11	12	1.37	4	2.63
13	*SSD*	1,290	3.79	92	10.51	26	17.11
14	*SSS*	28,392	83.34	328	37.49	31	20.39
15	*SSI*	882	2.59	100	11.43	16	10.53
16	*SID*	299	0.88	17	1.94	3	1.97
17	*SIS*	254	0.75	15	1.71	3	1.97
18	*SII*	142	0.42	17	1.94	2	1.32
19	*IDD*	136	0.40	11	1.26	4	2.63
20	*IDS*	107	0.31	11	1.26	2	1.32
21	*IDI*	38	0.11	17	1.94	1	0.66
22	*ISD*	232	0.68	18	2.06	7	4.61
23	*ISS*	307	0.90	16	1.83	3	1.97
24	*ISI*	133	0.39	27	3.09	4	2.63
25	*IID*	46	0.14	6	0.69	3	1.97
26	*IIS*	38	0.11	6	0.69	1	0.66
27	*III*	44	0.13	9	1.03	3	1.97
Totals		34,066		875		152	

Patterns of expression are denoted by a decrease, *D*, steady state, *S*, or increase, *I*, in each one of the three consecutive intervals, 10 to 20, 20 to 40 and 40 to 60 DAA, respectively. All analyses were performed at 0.01 FDR. For the 27 possible patterns, data for individual genes (Genes), Biological Processes (BP) and Metabolic Pathways (MP) are presented; in each case the number and percentage of the item following each pattern are presented.

The most common pattern of expression was represented by 28,392 genes (83.34*%*) that exhibited a consistent steady state mode of expression. In other words, these genes did not exhibit a significant change in expression between any of the consecutive time points (pattern 14−*SSS*). The remaining 5,674 genes (16.66%) exhibited a significant change in expression during at least one transition. The second- and third-most common patterns correspond to 13−*SSD*, represented by 1,290 genes (3.79%) and 15−*SSI* by 882 (2.59%) genes, respectively. Of particular interest, these two patterns represented by a total of 2,172 (6.38%) genes, were populated by genes showing significant changes in expression only in the third transition. This result suggests that the developmental period between 40 and 60 DAA was the most dynamic with respect to changes in the transcriptome. The fourth- and fifth-most common patterns were 5−*DSS*, represented by 537 genes (1.58%) and 23−*ISS*, represented by 307 (0.90%) genes, respectively. Patterns 5−*DSS* and 23−*ISS* were also characterized by genes showing significant changes of expression in only one transition, but in these cases, the relevant developmental period was between 10 and 20 DAA. Taken together, our results indicate that relatively large groups of chili genes showed the tendency to change their level of expression at only a single developmental stage and remain at this level until fruit maturity. The number of genes participating in patterns 13−*SSD*,15−*SSI*,5−*DSS* and 23−*ISS* includes a total of 3,016 genes (8.85%), which is more than half of the number of genes that exhibited a significant change in expression during at least one transition (5,674). In contrast, consider the patterns populated by genes whose expression changed significantly only in the middle transition (between 20 and 40 DAA). Examples of these patterns include 11−*SDS*, represented by 224 genes (0.66%) and 17−*SIS*, represented by 254 genes (0.75%). These patterns were relatively infrequent and populated by a total of 478 genes (1.41%). Patterns of consistent gene expression decrease (1−*DDD*) or increase (27−*III*) in each subsequent developmental interval were characterized by relatively few genes, 51 (0.15%) and 44 (0.13%), respectively. The least common pattern of gene expression observed was characterized by genes whose expression decreased in the first two transitions but increased in the third (3−*DDI*, represented by 11 (0.03%) genes). Additional file 2 includes gene identifiers, description, expression level, pattern and Q values for all genes detected in this study.

We also analyzed the global changes in gene expression during chili development by calculating the transition probabilities from one interval to the next. We begin by estimating the frequency of each transition during the first interval from 10 to 20 DAA (Table AF1-4 in Additional file 1). Then we calculated the conditional probabilities of change of state (*D*, *S* or *I*) from one transition to the next, assuming that a gene was selected at random. This allows the interpretation of the dynamic changes occurring during the transitions. For example, if a gene decreased its expression (*D*) during the interval 10 to 20 DAA, the most likely event is that its expression would remain at steady state *S* during the interval 20 to 40 DAA, and this probability was estimated to be 0.7339. All transition probabilities for genes whose expression were detected at a steady state level and then changed to a more active state, (from *S* to *I* or *S* to *D*) were larger for the transitions from 20 - 40 DAA to 40 - 60 DAA than from 10 - 20 DAA to 40 - 60 DAA. For example, the probability of changing from *S* to *D* was 0.0129 during the transition from 10 - 20 DAA to 20 - 40 DAA, but was four times more likely (0.0561) during the transition from 20 - 40 DAA to 40 - 60 DAA. This result suggests that the most active period for changes in expression frequencies occurred during the last period sampled, from 40 to 60 DAA.

**Figure 3:**
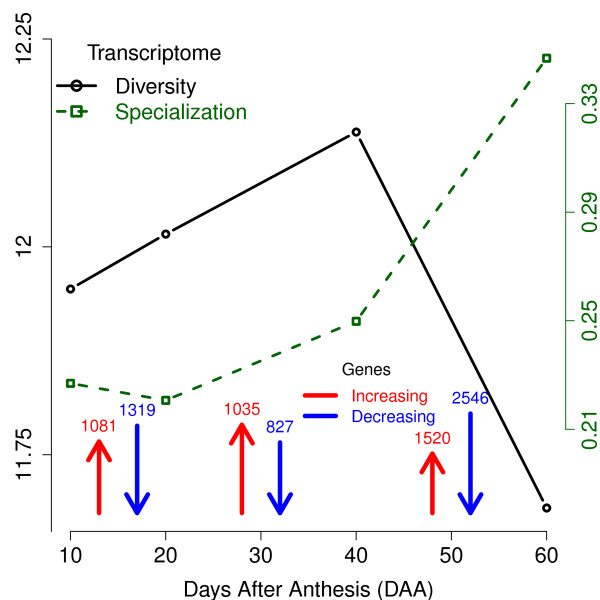
**Properties of the transcriptome and number of genes with significant change.** Plots present the estimated diversity (in bits) and specialization (in relative arbitrary units) of the chili pepper transcriptome during development. Colored arrows present the number of genes with significant changes per interval (0.01 FDR), either increasing (red arrows) or decreasing (blue arrows). The length of the arrows is proportional to the percentage of changes in the interval.

The number of genes exhibiting significant changes in expression during each interval of time is shown in Figure 3. The interval with the greatest number of genes showing significant changes in expression was between 40 and 60 DAA, with 1,520+2,546=4,066 changes, almost 50% of the total of significant changes detected in the three intervals (8,328 significant changes in 5,674 genes). Of these changes, 1,520 (37%) were increases in expression and 2,546 (63%) were decreases in expression, indicating that a larger percentage of genes were down-regulated during the last state of fruit development. In the first period, from 10 to 20 DAA, 2,400 significant changes were detected. 1,081 (45%) genes were up-regulated and 1,319 genes (55%) were down-regulated, while in the second interval (from 20 to 40 DAA) a smaller number of significant changes (1,862) were observed. During the second interval, 1,035 genes (56%) were up-regulated and 827 genes (44%) were down-regulated. In summary, development of the chili pepper fruit is characterized by two periods, from 10 to 20 and 20 to 40 DAA, where approximately 2,000 genes per period changed their pattern of expression. This was followed by a dynamic period (from 40 to 60 DAA) where more than 4,000 genes changed their expression pattern, and approximately 5/3 of these changes were decreases in expression. This may indicate a global down-regulation of metabolism marking the end of the maturation process. However, it should also be noted that the intervals between sampling points were not all equal in duration. Considering the global transcriptional changes on a per day basis, the first and third intervals appeared equally dynamic, exhibiting 2,400/10=240 and 4,066/20=203 changes in gene expression per day. During the intermediate period, between 20 to 40 DAA, only 1,862/20=93 changes in gene expression per day were observed.

We examined the expression patterns of genes encoding members of the xyloglucan transglucosylase/hydrolases (XTHs) family as examples of genes known to participate in fruit development and ripening. Genes belonging to the XTHs family where identified in our dataset using the results of the BLAST analysis. This family of enzymes is involved in xyloglucan endotransglucosylation and endohydrolysis and their respective genes are differentially expressed in mature-green and ripe tomato fruits [30,34]. The expression of the 11 members of the XTHs family exhibited the 7−*DID* pattern and a peak of expression at 40 DAA. However, the expression levels of individual members of this family was heterogeneous (see Figure AF1-1 and Table AF-5 in Additional file 1).

Classifying the patterns of expression of genes during chili pepper fruit development and ripening in the discrete space of 27 possible patterns allows for a more detailed analysis of the complex changes in the transcriptome. By considering only the *significant* differences in expression between sampling points, we can filter some of the noise produced by random differences in expression between biological replicates as well as excluding genes with very low expression in all states of development. On the other hand, pattern classification offers a complementary method to peruse the data, by focusing on genes that share the same patterns of expression. For example, within the subset of genes showing a continuous decrease in expression during development (pattern 1−*DDD*) are a number of orthologs of the Argonaute family. Argonaute proteins form an evolutionarily conserved family whose members silence gene expression in pathways such as RNA interference (RNAi) [35]. This suggests that the activity of the RNA-induced silencing complex (RISC) decreases during fruit development. Within the subset of genes whose expression increased in every subsequent developmental interval (pattern 27−*III*), we identified a senescence-associated gene, the chili pepper ortholog to Arabidopsis, “AT4G02380, senescence-associated gene 21”. The previous example of the XTH family also shows that examining the expression of related genes can lead to a better understanding of the underlying genetic and metabolic processes. An additional interesting avenue is to study the promoters of genes that share the same pattern of expression, with the aim of identifying specific sequence motifs involved in regulating genes according to these specific patterns [36]. This goal may be attainable when a chili pepper genome sequence becomes available.

### Changes in diversity and specialization of the transcriptome

To further examine the global changes in gene expression during chili fruit development, we estimated the diversity and specialization of our transcriptomes as well as the specificity of the genes detected [37]. The diversity of the transcriptome, *H*, is measured by the application of Shannon’s entropy formula to the set of estimated relative frequencies of expression of the genes. *H* is sensitive to both the number of expressed genes as well as to their frequencies, yielding higher values when the distribution of the frequencies is flatter. This scenario indicates that a larger content of information is passed from the nucleus to the protein synthesis machinery. We also estimated the specificity of each one of the genes. The specificity is zero if the corresponding gene is equally expressed in all the transcriptomes studied, and reach a maximum value when it is expressed exclusively at a single transcriptome (see [37] for details). Having the estimates of specificity for the genes we can estimate the specialization of the transcriptome, *δ*, as the weighted average of the specificity of the genes corresponding to that transcriptome. A larger value of *δ* indicates that, on average, more genes specific to the evaluated transcriptome are being expressed, i.e., the transcriptome is more “specialized” given that it express more specific genes. Figure 3 presents a plot for the diversity (*H*) and specialization (*δ*) of the transcriptomes we obtained during the development and ripening of chili pepper fruit. This figure also includes the number of significant changes in gene expression observed in each interval, discussed above. The diversity of the transcriptome increased from 10 DAA (*H*=11.95) to 40 DAA where it reaches its maximum value of *H*=12.14. Diversity then decreased abruptly in the interval from 40 to 60 DAA to its minimum value of *H*=11.69. Interestingly, there was no appreciable change of slope in the diversity increases between 10 to 20 and 20 to 40 DAA, indicating that the rate of increase in transcriptome diversity was constant from 10 to 40 DAA. This indicates that the point where genes were expressed at the most even frequencies was reached around 40 DAA, and, from that point of development, the transcriptome changed to a distribution in which genes were expressed at more variable frequencies (a less informative transcriptome). Another way to interpret the diversity of the transcriptome is to calculate the *number of effective genes*, $G=2^{H}$. This parameter represents the number of genes equally expressed required to produce a given diversity value of *H*. In contrast to *H*, which is given in logarithmic scale,  is given in additive genetic units and is thus easier to interpret. The estimated values of  were 3,954, 4,140, 4,507 and 3,295 for 10, 20, 40 and 60 DAA, respectively. If the maximum value of G=4,140 at 40 DAA is considered as 100%, the percentage-transformed values of  were 88, 92, 100 and 73% for 10, 20, 40 and 60 DAA respectively. This calculation makes it apparent that the  value diminished by 27% from 40 to 60 DAA. This abrupt change in the number of effective expressed genes indicates that the last developmental period examined (from 40 to 60 DAA) was the one characterized by the most profound changes in the transcriptome. This claim is equally supported by considering the number of genes differentially expressed during each transition (presented as arrows in Figure 3), while in intervals 10 to 20 and 20 to 40 DAA we see a more even ratio of around 50% in significant increases over the total of changes, (1,081+1,035)/(2,400+1,862)=2,116/4,262≈0.5, this ratio descends to 1,520/(1,520+2,546)≈0.37 in the last interval from 40 to 60 DAA, indicating a stronger down-regulation of the transcription machinery which results in a less diverse transcriptome at 60 DAA.

The specialization of the transcriptome, (*δ*), only slightly decreased at 20 DAA compared to 10 DAA, but at later time points it increased until reaching its maximum value at 60 DAA. This indicates that the transcriptome at 60 DAA was, on average, expressing more genes specific to that stage of development than at the other developmental time points. In contrast with the values for the number of genes detected at each time point (Figure 2), the specialization of the transcriptome gives a quantitative measure which responds to slight changes in the relative expression of the genes, allowing a better understanding of the global change experienced by these parameters.

Considering together the results for *H* and *δ* presented in Figure 3, we conclude that the chili transcriptome was most specialized and least diverse at 60 DAA and that the largest changes in the fruit transcriptome occurred in the period from 40 to 60 DAA. To provide some context for these observations, the human organs producing the least diverse and most specialized transcriptomes are the pancreas, salivary glands and stomach, whose functional role is to produce a relatively small number of metabolites in large quantities [37]. According to our analyses, mature chili fruits at 60 DAA are similar in that the transcriptome at this stage is less diverse and more specialized than the preceding developmental stages. This implies that more specific transcripts were produced in larger quantities than at the other stages sampled.

Figure AF1-2 in Additional file 1 presents the distribution of the gene specificity values [37] estimated in this study. In general, we observed a large proportion (25%) of *generalist* genes which were expressed in all four stages at approximately the same level, and a large proportion (18%) of highly specific genes that were almost exclusively expressed in only one of the stages of development. Combining the gene specificity parameters with information pertaining to differential expression of genes during each of the three periods of development represents a powerful tool to further investigate the biological roles of genes of interest.

### Grouping genes to gain biological knowledge

Grouping genes into categories has significant advantages to summarize gene expression information in extremely large datasets [38]. This approach reduces the dimensionality of the problem; instead of interpreting the expression patterns of thousands of genes, discrete groups of genes can be categorized in a variety of ways. This approach also increases statistical power, given that larger numbers of RNA-Seq reads can be taken into account in each comparison [4]. Models that explain transcriptional regulatory networks are an achievable goal, and the one developed for *E. coli*[39] is a good example. Eventually, we aim to develop models for eukaryotes that can explain and predict gene expression in different tissues and organs as a function of external and internal signals. By classifying patterns of gene expression into a smaller number of quantitative categories, it is possible to examine the tendencies of related groups of genes to coordinately change their levels of expression. There are currently a plethora of methods to analyze the expression of groups of genes (see for example [38,40]). In our study, we made use of a relatively simple measurement. By adding together all RNA-Seq reads mapping to genes participating in a particular category of interest, we obtained a quantitative parameter describing the expression of the category as a whole. This approach implies the reductionistic assumption that a category pattern of expression can be accurately represented as the sum of its parts. This is clearly not the case in categories characterized by genes in which some must be up-regulated and some must be down-regulated in order to participate in a particular biological activity. More sophisticated alternatives of analyzing genes in categories imply a better understanding of the function of each gene participating in a category. For example, certain transcription factors may be activated and a distinct set repressed. Thus, our approach of evaluating the sum of expression of genes participating in a category of interest is a crude, but necessary, first step in interpreting our expression results. This approach needs to be complemented with additional studies of the expression levels and functions associated with individual genes participating in each category.

In our study, we categorized chili pepper genes with sufficient annotation information into the Biological Process (BP) (from Gene Ontology [41]) terms and also into the Metabolic Pathways (MP) in which they participate. The latter classification scheme made use of the Kyoto Encyclopedia of Genes and Genomes (KEGG) [42]. For every chili gene that could be identified as an Arabidopsis ortholog, its corresponding BP term or MP was recorded. These classification schemes are somewhat redundant in that a single gene may participate in more than one BP or MP. To address this issue, we divided the estimated expression values (number of cDNA reads mapping to the gene) between the number of categories in which the gene has been annotated to participate and used this normalization for the grouping analysis. Thus, the total number of RNA-Seq reads considered for all genes was not altered, preserving the statistical power of the analysis (see Methods for details). Genes lacking sufficient annotation to participate in any of the BP or MP categories formed an “offset” category which was not tested but was included in the dataset. Even when the literature for the analysis of gene groups is very abundant (see for example [38,40]), to the best of our knowledge this is the first time that our particular approach to take into account the redundancy of the categories is reported.

### Genes grouped by Biological Process (BP)

In the BP category analysis, a total of 8,628 chili pepper genes were classified into 875 BP categories using the “slim” GO term (see Methods for details). The number and percentage of BP categories participating in each one of the 27 previously described global patterns of expression is presented in Table 2. Interestingly, the number of genes exhibiting a particular pattern of expression was highly correlated with the number of BPs showing the same pattern $\hat{\rho }=0.9458$ (${\hat{\rho }}^{2}=0.8945$); this suggest that not much relevant information was lost as a result of the grouping process and that the summarizing grouping procedure reflects the global behavior of gene expression. The most common pattern of expression for BP categories as well as for individual genes was 14−*SSS*. This pattern denotes genes whose expression did not change significantly over the sampling periods. The 14−*SSS* pattern includes 328 BP categories, which was 37.49% of the total. Expression patterns showing change only from 40 to 60 DAA (13−*SSD* and 15−*SSI*) comprised 10.51 and 11.43% of all BP categories, respectively. Examples of BP categories showing the 13−*SSD* pattern (a decrease in expression only during the third interval) were Histone methylation, Determination of bilateral symmetry, Secondary metabolic process, Meristem initiation, Pyrimidine ribonucleotide biosynthetic process and Meiosis. The 15−*SSI* pattern (a significant increase in expression only during the last interval) included BP categories such as Riboflavin biosynthesis, Defense response to fungus incompatible interaction, Cellular lipid metabolic process and Circadian rhythm. Translational elongation was the sole BP category characterized by the 1−*DDD* pattern (a decrease in expression during each subsequent interval). Nine BP categories exhibited the 27−*III* pattern, which is an increase in expression during each subsequent interval. These nine BP ontologies were Intracellular signal transduction, Amino acid transport, DNA-dependent negative regulation of transcription, Cellular membrane fusion, Response to abscisic acid stimulus, Response to ethylene stimulus, Negative regulation of endopeptidase activity, Regulation of proteolysis and Translational initiation. A table including results for each of the BP ontologies involved in our study is found in Additional file 3.

**Figure 4:**
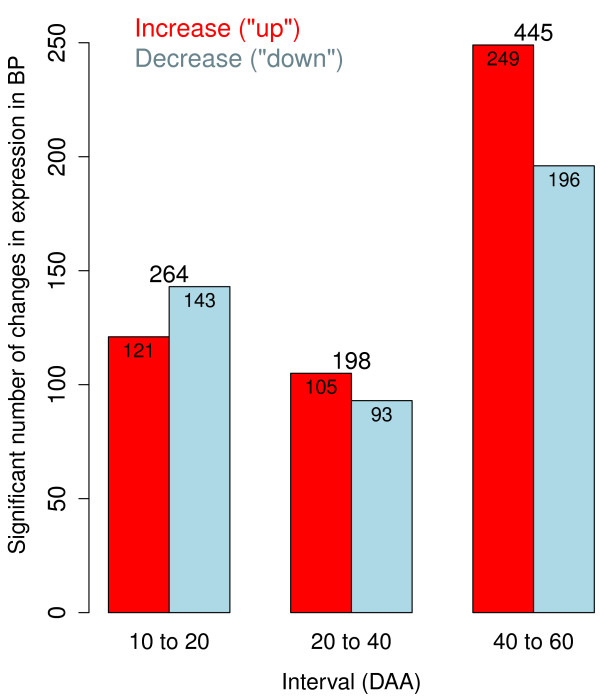
**Number of significant changes in expression of biological processes (BP) per interval.** The number of significant changes in expression (0.01 FDR) for groups of genes representing biological processes (BP) is plotted for each interval, between 10 and 20, 20 to 40 and 40 to 60 DAA. Red bars present the number of BP that increased expression while blue bars present the number of BP that decreased expression in the corresponding interval.

Histograms indicating the number of significant changes in BPs during each one of the developmental intervals are presented in Figure 4. From the total of 907 significant changes in the patterns of expression of the 875 BPs, the largest number of changes (445, or 49%) occurred in the last interval, from 40 to 60 DAA. This result is consistent with our analysis of individual genes, which also showed that the greatest number of genes was differentially expressed from 40 to 60 DAA (see Figure 3). Within these 445 changes in the behavior of the BP categories, a larger proportion was due to increases in expression (249 or 27%) than to decreases (196; 22%). This pattern is in contrast to the behavior of individual genes, where a larger proportion of genes showed decreases in expression in the last period. This implies that although a larger proportion of genes were down-regulated in the last period, a larger number of BPs were up-regulated or at least increased their net expression during this period of development. This inference is not complete because not all genes detected in the experiment could be categorized into BP ontologies. The number of significant changes in the net expression of BPs during the first two intervals (Figure 4: 10 to 20 and 20 to 40 DAA) was consistent with the expression results obtained for individual genes (Figure 3). In the first period a larger number of BPs decreased their net expression (143; 16%) while this pattern was reversed in the second period in which more BPs (105; 12%) increased their expression in comparison with the ones that decreased it (93; 10%). Consistent with our analysis of individual genes, the net changes in expression of genes participating in the BP categories indicates that the most active period of change in the transcriptome occurred during the last interval (from 40 to 60 DAA). However, because the length of the first interval was 10 days and the lengths of the other two intervals were 20 days, the rates of change in net expression of genes included in the BP ontologies were 264/10=26.4,198/20=9.9 and 445/20=22.3 per day for the intervals from 10 to 20, 20 to 40 and 40 to 60 DAA respectively. This result is also consistent with the rates of change in expression obtained for individual genes (see Figure 3).

Figure AF1-3 in Additional file 1 shows the expression of genes grouped into Fruit maturation BP as well as the expression of the grouped genes. Even when the analysis of the grouped expression shows a pattern of non significant changes (14−*SSS*), the pattern of all four genes forming this group shows a significant change in some of the intervals. In particular, the most influencing gene of the four (the one with largest expression and changes), the chili pepper orthologous of Arabidopsis AT1G47530, a MATE efflux family protein with functions in ripening, increases its expression significantly in the last two intervals, from 20 to 40 and 40 to 60 DAA. The fact that the grouped expression presents a pattern of non significant changes in this case is due to the changes in opposite directions from the genes forming the group. This illustrates the fact that it is important to examine not only the expression pattern of the group, but also of its individual components.

Figure AF1-4 in Additional file 1 shows the expression of 32 genes grouped into Developmental growth BP as well as the expression of the group as a whole. The pattern of change of this BP was significant at the three intervals, increasing in the first and decreasing in the following two intervals (pattern 19−*IDD*; green dashed line in Additional file 1: Figure AF1-4). The pattern 19−*IDD* of Developmental growth is consistent with what is known about the development of the chili pepper fruit, where the most active period of development occurs between 10 and 20 DAA to then decrease up to the fully mature state at 60 DAA (see Figure 1). In this case the graph of the individual behavior of the 32 individual genes grouped into Developmental growth BP is too complex to be directly interpreted. This shows the advantage of summarizing the expression of genes as groups.

### Genes grouped by Metabolic Pathway (MP)

Chili pepper genes (1,794) were grouped into 152 metabolic pathways (MPs) according to the classification of their corresponding Arabidopsis orthologs (see Methods). As for the BP ontologies, net changes in the expression of MPs were evaluated by summing the weighted expression of genes participating in each MP. The 27 possible patterns of change for the net expression of MP categories are shown in Table 2. The correlation between the patterns presented by genes considered individually and MP was estimated to be $\hat{\rho }=0.7209$ (${\hat{\rho }}^{2}=0.5196$) whereas the correlation between the patterns observed for BP and MP was estimated to be $\hat{\rho }=0.8636$ (${\hat{\rho }}^{2}=0.7458$). These measures indicate that there was greater concordance between the patterns presented by genes grouped into MP categories with genes grouped into BP categories than between genes grouped into MP categories with individual genes. As was previously observed for individual genes and genes grouped by BP, the most common pattern exhibited by genes grouped by MP was 14−*SSS*. 31 (20.39%) MP categories showed a steady-state level of net expression during chili development. As was the case for individual genes and BP categories, the next-most frequent patterns of change in the expression of MP categories were those exhibiting significant differences only in the third interval: 13−*SSD* with 26 (17.11%) MPs and 15−*SSI* with 16 (10.53%) MPs. These two patterns were populated by a total of 42 (27.64%) MPs, reaffirming the fact that the third interval (between 40 and 60 DAA) had the most profound transcriptional changes in our samples of chili pepper development. Full results pertaining to our pattern analyses of MP categories during chili development are shown in Additional file 3. A total of 121 (80%) of the MPs included in our study showed a significant change in net expression in at least one of the three intervals studied (Table 2). Only 2 MPs (Amino sugar and nucleotide sugar metabolism as well as Steroid biosynthesis) exhibited the pattern of significant decrease in each subsequent interval (pattern 1−*DDD*), while three MPs (Phenylpropanoid biosynthesis, Tryptophan metabolism and Tyrosine metabolism) exhibited the pattern of significant increase in net expression in each subsequent interval (pattern 27−*III*). This result suggests a crucial role for these metabolic processes during chili development and ripening, however, a recent metabolomic study of *Capsicum*[43] reports non significant changes in tyrosine during ripening while tryptophan was not detected, possibly by methodological shortcomings. On the other hand, the continuous increase in the expression of genes related with the phenylpropanoid biosynthesis during development could be related to the lignification of the fruit and also to the synthesis of capsaicinoids, as has been reported [44].

**Figure 5:**
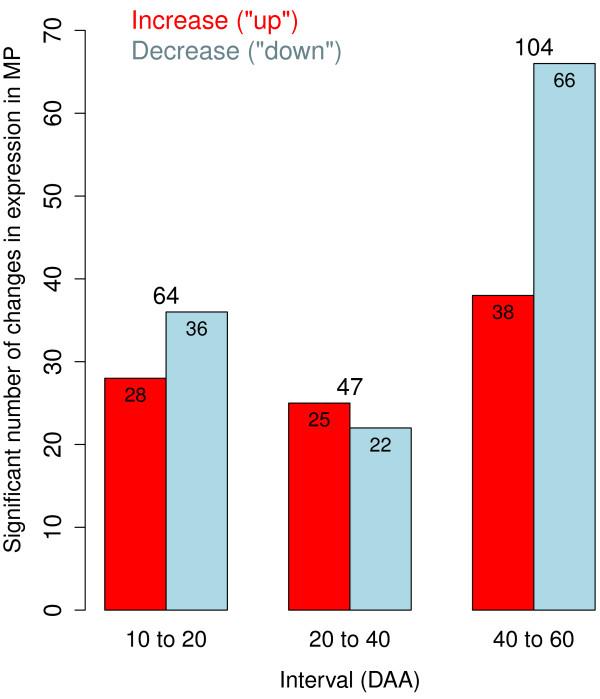
**Number of significant changes in expression of metabolic mathways (MP) per interval.** The number of significant changes in expression (0.01 FDR) for groups of genes representing metabolic pathway (MP) is plotted for each interval, between 10 and 20, 20 to 40 and 40 to 60 DAA. Red bars present the number of MP that increased expression while blue bars present the number of MP that decreased expression in the corresponding interval.

As previously discussed, these patterns observed of the MP categories reflect the weighted sum of RNA-Seq reads mapping to genes participating in the MPs. A more complete understanding of metabolic pathways playing important roles during chili development will require a careful analysis of the behavior of individual genes comprising the MP categories. The number of significant changes in the net expression of MPs during the three developmental intervals is summarized in Figure 5. The third interval (from 40 to 60 DAA) had the greatest number of significant changes in the net expression of MPs. During this interval, we calculated a total of 104 (48%) significant changes in MPs, with 38 (17.5%) showing increased and 66 (30.5%) showing decreased expression. This result is in accordance with our analysis of the expression of individual genes. The third period of development (from 40 to 60 DAA) had the greatest number of differentially expressed genes, most of which were down-regulated during this interval. Also, the tendencies observed in the analyses of individual genes during the first two intervals, from 10 to 20 and 20 to 40 DAA (Figure 3) were confirmed by the analyses performed by MP and presented in Figure 5. We found (36; 17%) MPs down-regulated in the first interval compared with (28; 13%) up-regulated MPs, while this tendency was reversed in the second interval where 22 MPs (10%) were down-regulated compared with 25 (12%) up-regulated. These observations could be due to the fact that the first period (between 10 and 20 DAA) occurs during the transition from flower to fruit, while in the second interval (between 20 to 40 DAA) new MP are activated. In the last period of maturation (between 40 to 60 DAA), metabolic activity decreases and thus a large number of MPs are down-regulated. Considering the four developmental time points together, 91 (42%) MPs were up-regulated and 124 (58%) were down-regulated. Similar to our calculations for the rate of change in the numbers of differentially expressed genes, the rate of change per day in MP expression was 64/10=6.4,47/20=2.35 and 104/20=5.2 changes per day for the intervals between 10 to 20, 20 to 40 and 40 to 60 DAA, respectively.

The expression patterns of genes associated with the capsaicinoid and ascorbic acid biosynthesis MP categories are shown in Additional file 1: Figures AF1-5 and AF1-6, respectively. Gene expression estimates for each gene participating in these categories are shown in Additional file 1: Tables AF1-6 and AF1-7, respectively. For the capsaicinoid biosynthesis pathway, the net expression pattern of the grouped genes participating in this pathway was 19−*IDD*, with a peak at 20 DAA. Six of the 13 individual genes grouped into this pathway also exhibited this pattern. This result is in agreement with previous studies of capsaicinoid accumulation in ‘Tampiqueño 74’ chili pepper fruits [9]. These pungent compounds begin to accumulate at 10 DAA, reach their maximum levels at 50 DAA and then decay [45]. With the aim of identifying genes involved in the biosynthesis of capsaicinoids Liu *et al*. performed an RNA-Seq study contrasting the placenta and pericarp of a highly pungent cultivar of *C. frutescens* L. [12]. Fruits were collected in the period of 15 to 25 DAA and hundreds of genes potentially regulating capsaicinoid biosynthesis were identified, which were predicted to be involved in microbody, peroxisome, fatty acid synthase activity, CoA-ligase activity, acyltransferase activity, transaminase activity, and phenylalanine metabolism, among other processes. In our study we varied the time period (DAA) studying whole fruits, and we identified groups of genes differentially expressed in time with peroxisome localization (23 genes) as well as genes involved in protein import into the peroxisome matrix (74 genes). In general, these genes tend to increase their expression as a function of time. In addition, genes involved in phenylalanine metabolism and CoA metabolism as well as acyltransferase or transaminase activities were found to be differentially expressed during chili development (see Additional files 2, 3, and 4 for details). Two genes recently shown to be involved in the capsaicinoids biosynthetic pathway, DHAD (dihydroxyacid dehydratase) and TD (thr deaminase) [13], were identified in our study. DHAD presented the expression pattern 13−*SSD*, while TD presented the pattern 1−*DDD*. A comparison of the results by Liu *et al*. for different parts of the fruit with the data reported here for changes in transcription activity trough time in the whole fruit provides a better understanding of the dynamics of the transcriptome during fruit development.

Genes categorized into the ascorbic acid biosynthesis pathway also exhibited the 19−*IDD* pattern. In this case, the pattern characterizing the whole group was driven by the expression of the GGP gene, which codify for a GDP-galactose phosphorylase [46] (see Figure AF1-6 and comments in Additional file 1). The results presented in Alós *et al*. [47] showing the relative expression of genes involved in the ascorbic acid pathway (Figure 3 of that reference) are highly correlated with the expression levels obtained here and presented in Additional file 1: Figure AF1-6 and Table AF1-7, even though the cultivars and methods employed are different. This suggests that the pattern for the expression of genes involved in the ascorbic acid pathway is consistent between cultivars when comparing equivalent states of development.

#### Carotenoid biosynthesis

Carotenoids are visual markers of chili pepper maturation. Color of the fruits may change from green to yellow, orange or red, depending on the type of carotenoids synthesized and accumulated by the fruits [48]. Nine genes grouped into the carotenoid biosynthesis MP exhibited the expression pattern 6−*DSI*, characterized by a significant decrease in net expression from 10 to 20 DAA, a steady state from 20 to 40 DAA and a significant increase between 40 to 60 DAA.

The expression patterns and levels for genes grouped into the carotenoid biosynthesis pathway are shown in Additional file 1: Table AF1-8 and Figure AF1-7, respectively. For eight of the nine genes grouped into this pathway, the expression pattern was characterized by a significant up-regulation between 40 and 60 DAA, where the color change from green to red in chili pepper fruits usually occurs (see Figure 1 and Additional file 1: Table AF1-8). Of these genes, the one with the largest change in expression was a gene encoding a capsanthin/capsorubin synthase. Expression of this gene increased markedly, from 6 transcripts per million (TPM) at 10 DAA to 15,206 TPM at 60 DAA (row 3 of Additional file 1: Table AF1-8, Panel A of Additional file 1: Figure AF1-7). Another gene with a large influence on the net expression behavior of the carotenoid biosynthetic pathway was a *β*-carotene hydroxylase (row 6 of Additional file 1: Table AF1-8, Panel A of Additional file 1: Figure AF1-7), which showed an expression change from 42 TPM at 10 DAA to 1,709 TPM at 60 DAA. Notably, none of these carotenoid biosynthesis genes were up-regulated during the first period from 10 to 20 DAA when chili fruits are still green. Our results confirm the findings of Romar *et al*. [49] showing that genes involved in the carotenoid biosynthetic pathway are not coregulated during chili fruit ripening, which is consistent with the hypothesis regarding differences in the expression of these genes. Ha *et al*. studied carotenoid accumulation and expression of genes involved in the carotenoid pathway in chili varieties with different levels of fully ripe color [50]. In that study the authors concluded that the expression levels of the phytoene synthase, phytoene desaturase, and capsanthin/capsorubin synthase genes are high in peppers with high levels of total carotenoids. These results are consistent with our findings, given that ‘Tampiqueño 74’ is a cultivar with strong red color at maturity (see Figure 1). Moreover, the results presented here add a more precise time frame for the expression of these genes (see Additional file 1: Table AF1-8 and Figure AF1-7).

#### Comparison of qRT-PCR vs. RNA-Seq results for genes related to carotenoid biosynthesis

**Figure 6:**
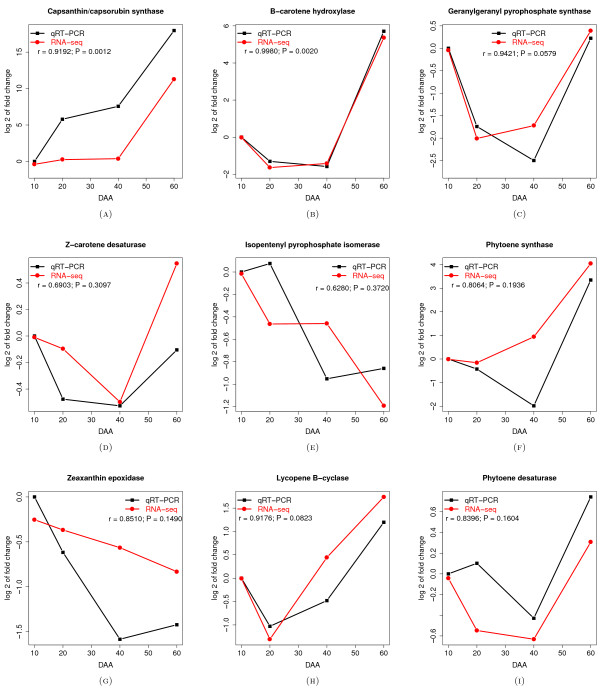
**Relative change of expression for nine genes related with carotenoid biosynthesis.***Log*_2_ of the fold change relative to the first point of development (10 DAA) is presented. Fold changes were originally estimated in the RNA-Seq experiment (red line) and validated by qRT-PCR (black line). In all cases the results are the average of two biological replicates. Confidence intervals for each point were too small to be represented in the plot. Pearson’s product moment correlation coefficient, r, and the P value for the hypothesis that *r*=0 are also presented for each gene tested. Panel **A** - Capsanthin/capsorubin synthase, **B** - *β*-carotene hydroxylase, **C** - Geranylgeranyl pyrophosphate synthase, **D** - Z-carotene desaturase, **E** - Isopentenyl pyrophosphate isomerase, **F** - Phytoene synthase, **G** - Zeaxanthin epoxidase, **H** - Lycopene B-cyclase and **I** - Phytoene desaturase.

In order to validate the chili pepper fruit transcriptome RNA-Seq results, we conducted a qRT-PCR analysis of the nine above mentioned carotenoid biosynthesis genes (Table AF1-8 in Additional file 1). The expression of each of the nine genes was calculated at each developmental time point using the $2^{-\Delta \Delta C_{T}}$ method [51] and expressed as a *log*_2_ fold-change relative to the level of expression determined at 10 DAA. Figure 6 presents the *log*_2_ fold-change in expression estimated using both RNA-Seq and qRT-PCR methods as well as the Pearson’s correlation (*r*) between the fold-changes obtained from each method for each gene. The values of *r* for the nine genes ranged from a minimum of 0.6280 (for isopentenyl pyrophosphate isomerase) to a maximum of 0.9980 (for *β*-carotene hydroxylase), with a mean value of 0.8436. Thus, the expression estimates obtained using the two methods were largely in agreement. We evaluated the concordance between these two methods by comparing their ability to discriminate the tendencies of genes to significantly increase or decrease in expression according to the previously established 27 patterns. For three genes (capsanthin/capsorubin sythase, zeta-carotene/neurosporene desaturase and lycopene *β*-cyclase) (panels A, D and H in Figure 6) both methods identified the same trend during all three intervals. For 5 genes (panels B, C, F, G and I in Figure 6), both methods identified the same trend in two of three intervals. Only for the isopentenyl pyrophosphate isomerase-encoding gene (panel E in Figure 6) there was a complete discordance between the two methods. The standard deviation values for the expression of the isopentenyl pyrophosphate isomerase-encoding gene in the RNA-Seq analysis (Additional file 1: Table AF1-8) were the largest among all 9 genes related to carotenoid biosynthesis. We speculate that expression of this gene has a larger intrinsic variation than the other genes considered, and this can explain both the low level of correlation (*r*=0.6280) as well as the lack of concordance between the RNA-Seq and qRT-PCR estimations.

Our qRT-PCR analysis also confirmed the finding that genes pertaining to carotenoid biosynthesis were most abundantly expressed during the last period of fruit maturation, reaching their maximum levels at 60 DAA.

## Conclusions

This study presents a detailed analysis of gene expression in chili pepper fruit at four stages of development. We estimated that our RNA-Seq transcriptomes may be missing approximately 6,000 genes expressed at very low levels and that the scaled number of mRNA molecules in the samples were about the same sizes as the number of cDNA reads obtained. We analyzed the expression patterns of individual genes, as well as groups of genes categorized into Biological Processes (BP) ontologies and Metabolic Pathways (MP). The most prevalent pattern was for individual genes as well as the BP and MP groupings to change their behavior only during the third interval, from 40 to 60 DAA. In this interval, down-regulated genes were more prevalent, marking the end of the fruit maturation and the starting point of senescence. We also concluded that transcriptome diversity was at its maximum at 40 DAA and that the transcriptome of the mature fruit at 60 DAA was the most specialized and least diverse of the transcriptomes studied.

We demonstrated that by grouping genes and studying the pattern of expression of the groups as well as the individual components, it was possible to gain insight into the behavior of the whole system. By using this approach we inferred, for example, that the most abundant expression of genes related to capsaicinoid and ascorbic acid biosynthesis occurred at 20 DAA, while for genes related to carotenoid biosynthesis the maximum was estimated at 60 DAA.

## Methods

### Biological material and RNA extraction

*Capsicum annuum* Serrano ‘Tampiqueño 74’ seeds were germinated as previously described [21] and the seedlings were cultivated until maturity under greenhouse conditions in a completely randomized experimental design at Cinvestav-Unidad Irapuato (Guanajuato, México). The plants were grown during the spring and summer, and individual flowers were tagged at anthesis. Chili pepper fruits were randomly collected from different plants at 10, 20, 40 and 60 DAA. After sampling, the fruits were cleaned with ethanol immediately frozen in liquid nitrogen and stored at -80°C until further use. For total RNA extractions, 10 fruits at the 10 DAA developmental stage and 5 fruits from each of the 20, 40 and 60 DAA stages were randomly selected from the pool of all harvested fruits. Whole fruits (pericarp, placenta and seeds) were ground in liquid nitrogen with a mortar and pestle to form a fine uniform powder. Samples were mixed vigorously and 100 mg aliquots were measured for each RNA extraction. The process was repeated with different sets of fruits in order to obtain two independent biological replicates at each developmental stage. A NucleoSpin RNA Plant kit (Macherey-Nagel) was used for total RNA extraction and contaminating genomic DNA was removed by DNase I (Macherey-Nagel) treatment following the manufacturers’ protocols.

Total RNA concentration was quantified using a NanoDrop ND-1000 spectrophotometer (Nano-Drop, Wilmington, DE, USA) and RNA quality was evaluated by gel electrophoresis on 1.0% denaturing agarose gels. In addition, aliquots of RNA were run on an Agilent 2100 Bioanalyzer using RNA 6000 chips (Agilent, Santa Clara, CA, USA) to test the RNA integrity number (RIN). All eight samples had RIN values higher than 8.7. Thirty *μ*g of total RNA from each of the eight samples (two biological replicates of four fruit developmental stages) was used for cDNA library preparation.

### Library construction and sequencing

The eight total RNA samples (two biological replicates from chili pepper fruits at 10, 20, 40 and 60 DAA) were prepared for RNA-Seq using the Illumina TruSeq RNA Sample Preparation v2 Guide following the manufacturer’s instructions. Briefly, mRNA was purified from 20 *μ*g of total RNA using poly-T oligo-attached magnetic beads using two rounds of purification. During the second elution of the poly-A RNA, mRNA was fragmented using divalent cations under elevated temperature and primed for cDNA synthesis. The cleaved RNA fragments were primed with random hexamers and reverse transcribed into single-stranded cDNA using reverse transcriptase. In the next step, the RNA template was removed and the complementary cDNA strand was synthesized using RNAse H and DNA polymerase I, respectively.

The overhangs that resulted from fragmentation were polished into blunt ends using an End Repair Mix (consisting of T4 DNA polymerase, Klenow fragment and T4 polynucleotide kinase). A single *T* nucleotide was added on the 3’ end of the adapter for ligating the adapter to the cDNA fragments. Indexing adapters were ligated to the ends of the cDNAs using T4 DNA ligase, preparing them for hybridization onto a flow cell. Finally, the DNA fragments with adapter molecules at both ends were amplified by PCR to enrich the amount of DNA in the library. PCR was performed with a PCR primer cocktail that anneals to the ends of the adapters. Quantity and quality of the DNA libraries was assessed using Agilent DNA-1000 chips on an Agilent 2100 Bioanalyzer.

The 8 cDNA libraries were pooled and simultaneously sequenced from both 5’ and 3’ ends using the Illumina MiSeq *®*; System platform according to the manufacturer’s instructions. We performed three sequencing runs (technical replicates) with the aim of increase the sequencing depth. 150 bp paired-end reads were obtained in each sequencing run (see Table AF1-1 in Additional file 1). Fluorescent image processing, base-calling and quality value calculations for each of the three runs were performed using Illumina MiSeq Control Software. Data were deposited at the NCBI (GEO database under series record GSE54123).

### Quality filtering, *de novo* assembly and remapping

Before assembly, the raw reads were filtered using PRINSEQ 0.20.3 software to obtain high-quality reads lacking adaptor sequences or ambiguous nucleotides. *De novo* assembly of the trimmed reads was performed using Trinity [3] (release 20121005) using DIAG (Data Intensive Academic Grid facilities) [52] (See Additional file 1 for details). The total number of sequences obtained and their characteristics are presented in Table AF1-2 in Additional file 1. For remapping sequence reads to the assembled contigs and transcript quantification of 45,505 genes and 99,487 isoforms assembled, we used RSEM [53] version 1.2.0 software (see Table AF1-2 in Additional file 1 for details).

### Sequence annotation

For annotation purposes, only the principal isoform of each contig generated by the Trinity assembler was used. Various BLAST databases were sequentially queried with the chili pepper fruit sequences to obtain the most likely orthologs. Details pertaining to this process as well as the results of the BLAST queries are presented in Table AF1-3 in Additional file 1.

Blast2GO [54] was also used to obtain GO biological process information from BLASTx TAIR 10 hits using default parameters for the annotation rule. Metabolic pathway (MP) assignments were carried out based on the KEGG database [42] using the KAAS server automatic annotator [55].

The data for the capsaicinoid and ascorbic acid pathways (which are not present in KEGG) were obtained by manual curation of the data. This procedure consisted in identifying in our dataset the chili pepper sequences coding for each one of the enzymes taking part in each one of the two pathways (see [9,45,48] for the capsaicinoid pathway and [46] for the ascorbic acid pathway). We used the relevant polipeptide sequences from the GenBank database [56] (see Additional file 1 for details). The statistical analyses of these two pathways was performed as described for all the other gene groups.

### qRT-PCR analysis

To confirm our RNA-Seq results, nine genes associated with the carotenoid pathway [10] were chosen for expression validation using qRT-PCR with gene specific primers. Primers were designed with primer 3 plus software [57] and are listed in Additional file 5. Total RNA was extracted using a NucleoSpin RNA Plant kit (Macherey-Nagel) from independent samples of whole fruits collected at 10, 20, 40 and 60 DAA. Total RNA (1 *μ*g per sample) was digested with DNAse I (Invitrogen, Carlsbad, CA) to remove DNA. RNA concentration was measured using a ND-1000 spectrophotometer (NanoDrop products, Wilmington, DE) and 500 ng of total RNA per sample was reverse transcribed using SuperScript III Reverse Transcriptase (Invitrogen). All qRT-PCR reactions were performed using an ABI 7500 Real Time System (Applied Biosystems) using ubiquitin (*Capsicum annuum* hexameric polyubiquitin 6PU11, ubi11; GenBank accession AY496112) and elongation factor (*Capsicum chinenseEF*1*α* mRNA for elongation factor 1 *α*; GenBank accession AB275381) genes as the internal controls. Each 20 *μ*L reaction was composed of 1 *μ*L cDNA (100 ng/uL), 2 *μ*L of primer mix (10 mM of each forward and reverse primer), 8 *μ*L of sterile water and 10 *μ*L of SYBR Green PCR Master Mix reagent (Applied Biosystems). Reactions were performed with an initial incubation at 50°C for 2 min and at 95°C for 10 min, and then cycled at 95°C for 15 s, and 60°C for 60 s for 40 cycles. Duplicates (biological replicates) from independent RNA extractions of each state of development were performed. Relative gene expression was calculated using the $2^{-\Delta \Delta C_{T}}$ method [51]. The qRT-PCR results are presented as fold-changes in gene expression relative to the 10 DAA sample (Figure 6).

### Statistical analyses

Data resulting from sequencing, assembly and annotation procedures were collected into a MySQL Ⓒ relational database (Server version 5.1.49). Expression data for each contig was obtained as the number of reads remapped by the RSEM software [53] version 1.2.0 (see Table AF1-2 in Additional file 1 for details). No correction for transcript length was applied, given that we are always comparing the same gene under different conditions (stage of development of the chili pepper fruit) [28]. All statistical analyses were performed in R [31] version 2.15.3 (2013-03-01). Expression data for the 42,401 contigs in the eight sequenced libraries (see Table AF1-2 in Additional file 1) was handled by summing the numbers of reads that mapped to the contigs that shared the same identifier from the BLAST analyses. In other words, we considered contigs with the same BLAST identifier to represent the same gene. This resulted in a data matrix with 34,066 rows (called here “chili pepper genes”, even if some cases they were identified with an orthologous gene from other specie) and eight columns (libraries; see Table AF1-3 in Additional file 1). To estimate the true number of genes expressed in each transcriptome (Table 1) we used equations described in Chao [26] and Changjiang *et al.*[27]. To estimate the scaled number of mRNA molecules, $\hat{M}$, presented in Table 1, we used Equation (8) in Good [25].

The R package edgeR[28] was used to evaluate differential gene expression (DGE) between adjoining intervals during development (between 10 to 20, 20 to 40 and 40 to 60 DAA). Other statistical contrasts involving not adjoining intervals were not performed because we considered that all relevant information about the changes of expression is already present in the analysis of adjoining intervals. Briefly, for each contrast (adjoining interval) we entered the matrix containing the data (counts of the number of reads for each gene in each library) using the DGEList function, estimated common and tag-wise dispersion and performed the exact test via the exactTest function. P values resulting from the exact test were then fed into the qvalue function [33] with default parameters, except that we set fdr.level=0.01 to obtain a false discovery rate of 1%. The resulting Q values were used to classify the genes into the patterns of expression presented in Table 2. All information resulting from the analyses was stored in the MySQL relational database for interpretation and complete results are presented in Additional file 2.

To perform differential expression analyses between groups of genes associated with BP or MP of interest, we collapsed the original data matrix (34,066 rows and eight columns) into transformed matrices that additively reflected the expression of each of the gene groups. To achieve this, we first selected and classified all genes with their corresponding category (BP or MP). Given that some of the categories are redundant, i.e., a gene can belong to more than one BP or MP, the numbers of reads per gene (tag counts) for genes belonging to more than one category were divided between the number of genes in the corresponding category. For example, if gene “*a*” was classified into 5 BP categories, the original number of gene tags for gene “*a*” in each library was divided by 5. Having the weighted expression for all genes belonging to at least one category we added the weighted expression of genes in each category to obtain the final category expression. The expression (number of reads) of all genes which were not classified into a category were added together to form the last row of the matrix of group expression. This last row is taken as an offset and was not tested. It is important to notice that this procedure conserve the amount of statistical evidence existent in the original matrix, given that the transformed matrices have the same total of reads (sum per column) than the original matrix. Transformed matrices were subjected to the same statistical procedure than the one performed for single genes (see previous paragraph), obtaining the Q values corresponding to each pair of neighboring intervals. All information resulting from these analyses was stored in the MySQL relational database for interpretation and complete results are presented in Additional file 3 for BP and in Additional file 4 for MP categories.

## Availability of supporting data

The data sets supporting the results of this article are included within the article (and its additional files). Raw reads for the eight libraries sequenced are available in the NCBI, Gene Expression Omnibus (GEO) repository, http://www.ncbi.nlm.nih.gov/geo/query/acc.cgi?acc=GSE54123.

## Competing interests

The authors declare that they have no competing interests.

## Authors’ contributions

LAM-L performed sample collection, library preparation, gene annotation as well as qRT- PCR analyses, participated in bioinformatics and statistical analyses and drafted the Methods Section; NO-A conceived the research, interpreted the results and shared in manuscript preparation; OM, shared data interpretation directed the bioinformatics pipelines, designed and performed statistical analyses and wrote the manuscript. All authors read and approved the final manuscript.

## Supplementary Material

Additional file 1 **Additional Tables and Figures.** Tables and Figures from this file are referred as “AF1-#”, where “#” is the corresponding number both, in the main text as well as in Additional file 1. Click here for file

Additional file 2 Excel file containing the identifiers, descriptions, pattern of expression, Q values and expression (in TPM) for all the chili genes in this study. Click here for file

Additional file 3 Excel file containing the identifiers, number of genes, Biological Processes (BP’s), pattern of expression, Q values and weighted expression for all the biological processes (BP) in this study. Click here for file

Additional file 4 Excel file containing the identifiers, Metabolic Pathways (MP’s), number of genes, pattern of expression, Q values and weighted expression for all the metabolic pathways (MP) in this study. Click here for file

Additional file 5 **Gene-specific primers for the qRT-PCR analysis.** Primers designed for the qRT-PCR analysis of 9 genes related to carotenoid biosynthesis. Click here for file
