# Supplementary material for: Dynamics of the chili pepper transcriptome during fruit development
Source: BMC Genomics. 2014 Feb 21;15:143. doi: 10.1186/1471-2164-15-143 (PMC3936869; doi:10.1186/1471-2164-15-143)
Supplement: Additional file 1 — Additional Tables and Figures. Tables and Figures from this file are referred as “AF1-#”, where “#” is the corresponding number both, in the main text as well as in Additional file 1. [file 1471-2164-15-143-S1.pdf]

# - ADDITIONAL FILE 1 - ADDITIONAL TABLES AND FIGURES FOR "DYNAMICS OF THE CHILI PEPPER TRANSCRIPTOME DURING FRUIT DEVELOPMENT"

LUIS A MARTÍNEZ-LÓPEZ, NEFTALÍ OCHOA-ALEJO AND OCTAVIO MARTÍNEZ*
*CORRESPONDING AUTHOR OMARTINE@LANGEBIO.CINVESTAV.MX

Here we present and discuss additional Tables and Figures; Material in this Additional File are referred to in the main text as "AF1-#", where "#" is the corresponding number for table or figure.

1. Sequencing, assembly and remapping

Table AF1-1 presents the number of raw and filtered reads obtained from sequencing the 8 cDNA libraries constructed. The total number of bases obtained in each library is presented. The row "Mapped" in Table AF1-1 indicates the number of reads and bases that were successfully mapped to the contigs obtained in the assembly.

Table AF1-1. Summary of reads and bases obtained from each stage of chili pepper development sampled. Reads are given in millions (M) and bases are given in Megabases (Mb).

Number of	Status	Days After Anthesis (DAA) 10	20	40	60	Total
Reads (M)	Raw	3.83	4.25	4.24	4.55	16.87
	Filtered	3.53	3.93	3.92	4.17	15.55
	Mapped	1.86	2.07	1.78	2.31	8.02
Bases (Mb)	Raw	566.33	629.20	617.26	673.01	2,485.80
	Filtered	521.61	580.46	578.39	617.40	2,297.86
	Mapped	279.72	309.78	267.16	345.99	1,202.67

1.1. **Quality filtering.** Before assembly, the raw reads were filtered using PRINSEQ 0.20.3 software to obtain high-quality reads. Duplicated sequences, reads containing more than 2% of ambiguous bases ("N"), low complexity reads with an entropy value of less than 70 and low-quality reads exhibiting a mean quality score Q-value ≤ 25 were eliminated from further analyses. The Q-value is the quality score assigned to each base by the

Illumina base-caller in the Illumina MiSeq Control Software, which is similar to a Phred score. The command-line parameters for PRINSEQ 0.20.3 were:

`−fastq1.fq − fastq22.fq −out_format3 −min_qual_mean25 −ns_max_p2`

`−noniupac − derep1 −lc_methodentropy −lc_threshold70.`

1.2. **De novo reference sequence assembly.** De novo assembly of the filtered reads was performed using Trinity [9] (release 20121005) using the DIAG (Data Intensive Academic Grid facilities) [3] with 48 G RAM per node and using 32 Gb for the Jellyfish step [15]. The size of the k-mers used in Trinity was 25 and the rest of the assembly parameters were set as default. The command-line parameters used in the assembly were:

`− − seqTypefq − − left1.fq − − right2.fq − −CPU8 − −JM32G − − no_cleanup`

Trinity software implements the Bruijn graph algorithm and a modular strategy. Briefly, the filtered reads were assembled into unique transcript sequences using the Inchworm module. In the next step, the Chrysalis module clustered the Inchworm contigs into and constructed complete de Bruijn graphs for each cluster. In the third step, the Butterfly module processed the individual graphs in parallel, reported full-length transcripts for alternatively spliced isoforms and teased apart transcripts that correspond to paralogous genes. Table AF1-2 presents the number of contigs obtained as well as summary statistics of the assembly.

Table AF1-2. Summary for *the novo* transcriptome assembly

Statistic	Value
All isoforms	99,487
Contigs	45,505
Total base pairs	122,671,190
Mean sequence length	$1,233.04 \pm 1,122.16$ bp
Mean GC content	38.79 ± 3.87
N_{50} contig size	1,903 bp
N_{75} contig size	1,098 bp
Mapping ratio (average in %)	65.68
Contigs with expression	42,401

1.3. **Remapping and quantification.** RSEM [14] version 1.2.0 was used for remapping the reads to the assembled contigs and transcript quantification of the 45,505 contigs and 99,487 isoforms assembled with Trinity (see Table AF1-2). This software estimated the expression levels taking into account read mapping uncertainty [14] using an Expectation-Maximization algorithm. Briefly, this process consisted of two steps. First, a set of reference transcript sequences was generated and pre-processed by the script rsem-prepare-reference using Bowtie [13] version 0.12.7 for constructing the indices. The following default parameters were used:

`rsem − prepare − referencename_assembled_file.fastacustom_reference_name`

Secondly, RNA-Seq filtered reads were aligned to the reference contigs and the resulting alignments were used to estimate transcript abundances. We used the following default parameters:

`rsem − calculate − expression − −paired − end`

`name_fastq_left.fastqname_fastq_right.fastq`

`custom_reference_namecustom_output_name`.

The script rsem-calculated-expression used Bowtie for the alignment with the following parameters:

`bowtie − q − − phred33 − quals − n2 −e99999999 − l25 −I1 − X1000 − p1 − a − m200 − S`

`custom_name − 1 left.fastq − 2 right.fastq`.

After the alignment of reads, RSEM computed maximum-likelihood abundance estimates using the Expectation-Maximization algorithm for its statistical model.

In order to obtain assembly statistics for the ratio of the number of reads that mapped back to contigs (mapping ratio), we used the perl script "alignReads" supplied with Trinity's utilities and was run with the following parameters:

alignReads.pl − − leftleft$_n$ame$_f$ile.fastq − − rightright$_n$ame$_f$ile.fastq

`−seqTypefq − − targetname_assembled_file.fasta − − alignerbowtie`

`− − retain_intermediate_files`.

The name-sorted file output was used to assess the number of reads that were found to align to contigs as properly paired, individually, or as improper pairs. For that, we used the perl script

SAM_nameSorted_to_uniq_count_stats.pl supplied within the Trinity tools for downstream analysis.

2. Sequence annotation

For annotation purposes we used only the principal isoform of each one of the contigs produced by the Trinity assembler. We used the contigs to query various databases using a local installation of NCBI BLAST version 2.2.26 [1]. The databases searched were, in order of priority, Arabidopsis polypeptides (TAIR 10 database [10]), the NCBI polypeptide reference sequences (RefSeq 56, only plants[19]), the polypeptides of tomato (ITAG 2.3 [11]), nucleotide sequences from *Capsicum annum* Capsicum DB [7],and Capsicum assemblies from PlantGDB [4]. As a first step, all chili pepper sequences were used as queries versus TAIR 10. Sequences with a significant hit ($E \leq 1e - 6$) were considered as identified and

eliminated from the query list. The remaining sequences were used as queries versus RefSeq 56 and, again, the ones with a significant hit ($E \leq 1e-6$) were considered as identified. Unidentified sequences were queried versus the nucleotide databases Capsicum DB and were screened with a less stringent E value of 1. All results were stored in our own relational database and Table AF1-3 summarizes the results of this annotation process.

To further annotate our chili pepper gene assemblies, Blast2GO [2] software v2.6.1 was used to obtain GO Biological Process information from BLASTx TAIR 10 hits using default parameters for the annotation rule. These default parameters were: E-Value Hit Filter $=1.0E-6$, Annotation Cut-Off=55, GO-Weight=5, Hsp-HitCoverage CutOff =0. Also, the GOslim function (goslim_plant.obo), implemented in Blast2GO was used to obtain specific GO terms by means of a plant-specific reduced version of the Gene Ontology[6] for the Biological Process category used in this study. Metabolic Pathway (MP) assignments were carried out based on the KEGG database [12] using the KAAS server automatic annotator [18] with a bit score threshold of 60 (default), BBH (bi-directional best hit) like assignment method. Arabidopsis thaliana and Oriza sativa were chosen for the BLAST genes dataset. Given that the assignment of the KEGG gene hits to the KEGG pathways by the KAAS server was general and not plant-specific, we performed a manual curation for plant-specific pathways. The capsaicinoid and ascorbic acid pathways, which are not available at KEGG, were manually curated by identifying the genes involved in each one of these MP. With that aim we downloaded the sequences reported for each enzyme involved in each pathway at the GeneBank nucleotide database [5], and used them as queries via BLAST to our transcriptome assembly, identifying genes encoding enzymes relevant to each pathway. All results were added to our relational database.

Table AF1-3. Summary of BLAST annotation of the chili contigs.

Queried Database	Chili contigs		Identifiers	
	Number	Percentage	Number	Percentage
TAIR 10 (Arabidopsis)	19,018	44.85	12,410	70.74
REFSEQ 56 (only plants)	1,687	3.98	1,051	5.99
ITAG 2.3 (Tomato)	4,939	11.65	3,848	21.94
Capsicum DB (Chili pepper)	233	0.55	233	1.33
Total Identified	25,877	61.03	17,542	100.00
Total not Identified	16,524	38.97	0	0.00
Total contigs	42,401	100.00	–	–
Estimated number of chili genes:		$16,524 + 17,542 = 34,066$		

2.1. **Transition frequencies during development.** To analyze changes in gene expression during the development of chili fruit, we calculated transition frequencies from one interval to the next. This was accomplished by estimating the conditional probabilities of a change of state in the genes through the different periods of time, assuming that a gene is selected at random from all genes detected in the experiment. Table AF1-4 presents

such frequencies. The first row of Table AF1-4 shows the frequencies of genes at interval 10 to 20 DAA. The majority of the genes, 0.9295 in relative frequency, were estimated to be in a steady state, i.e. they did not change significantly in expression between 10 and 20 DAA. In rows 2 to 4 of Table AF1-4 we examined the probabilities that a gene was found to have a particular behavior in the second interval (20 to 40 DAA), given that it was in a particular state in the previous interval (10 to 20 DAA). For example, we observed that, if a gene was down-regulated D during the interval 10 to 20 DAA, the most likely event was to find it at a steady state S in the interval 20 to 40 DAA, and this probability was estimated to be 0.7339. Rows 5 to 7 of Table AF1-4 show the transition frequencies for the interval 40 to 60 DAA given that the behavior of the gene during the interval 20 to 40 DAA was known. The probability of finding a gene at steady state S, during the interval 40 to 60 DAA given that it was down-regulated D, at 20 to 40 DAA is only 0.4933 and not 0.7339, as was the case for the transition from the previous interval. All transition probabilities that go from a steady state to an active state (from $S \Rightarrow I$ or $S \Rightarrow D$) were larger for the transitions from 20-40 DAA to 40-60 DAA than from 10-20 DAA to 40-60 DAA. For example, the probability of change from the state S to D is only 0.0129 from 10-20 to 20-40 while it is more than 4 times larger, 0.0561, from 20-40 to 40-60 DAA. This implies that the most active period for changes in expression frequencies occurs in the last period sampled, from 40 to 60 DAA.

Table AF1-4. Estimated transition frequencies between states (D - Decreasing, S - Steady, I - Increasing) for individual genes.

	Frequency at 10 to 20		
	D	S	I
	0.0387	0.9295	0.0318
State at	Given that at 10 to 20 it was		
20 to 40	D	S	I
D	0.1054	0.0129	0.2599
S	0.7339	0.9652	0.6216
I	0.1607	0.0219	0.1184
State at	Given that at 20 to 40 it was		
40 to 60	D	S	I
D	0.4027	0.0561	0.3913
S	0.4933	0.9078	0.3440
I	0.1040	0.0360	0.2647

2.2. **Changes in gene expression among XTH family members.** As an example of pertinent changes in gene expression during the development of chili pepper, Figure AF1-1 shows the expression levels of 11 members of the Xyloglucan transglucosylase/hydrolase (XTH) family. This family of genes is differentially expressed between the mature-green and ripe stages in tomato fruits [17, 23]. In total, we identified 16 chili pepper contigs with

high similarity to members of the XTH family. Of these, 11 exhibited significant changes in expression during at least one interval and an analysis of these genes is presented in Figure AF1-1 as well as Table AF1-5. The averaged pattern of expression of the 11 members of the XTH family was typically $7 - DID$, with a peak of expression at 40 DAA (see mean expression in Figure AF1-1 and Table AF1-5). This pattern of expression, obtained as a result of our strategy of grouping genes, was most obvious for 3 genes, particularly the ortholog of AT5G57560.1, which was expressed at a level of 560 TPM at 10 and 40 DAA. Two groups comprised of two genes each presented the patterns $13 - SSD$ and $16 - SID$, while the remaining five genes presented other patterns of expression. The only gene whose expression increased during each subsequent time point (pattern $27 - III$) was the ortholog of AT1G32170.1 (xyloglucan endotransglucosylase/hydrolase 30) which was expressed at 8 TPM at 10 DAA and increased to 246 TPM at 60 DAA. This expression pattern was detected in only 44 genes in the entire transcriptome dataset, 0.13% of the total (see Table 2 in main text).

Table AF1-5. Expression patterns of chili pepper genes orthologous to *Xyloglucan endotransglucosylase/hydrolase* (XTH family)

Pattern	Orthologous identifier	Expression in TPM 10 DAA	20 DAA	40 DAA	60 DAA
4-DSD	AT4G14130.1	55	28	13	0
7-DID	AT5G57560.1	566	19	559	303
7-DID	AT4G03210.1	241	116	344	0
7-DID	AT3G23730.1	101	44	129	3
11-SDS	AT2G36870.1	41	40	7	0
13-SSD	AT5G13870.1	256	230	203	70
13-SSD	AT1G11545.1	26	17	35	0
16-SID	AT4G30270.1	13	6	77	13
16-SID	AT5G57550.1	0	0	21	0
25-IID	AT4G37800.1	32	134	250	1
27-III	AT1G32170.1	8	69	114	246
7-DID	Mean	122	64	159	58
Standard deviation		172	70	171	110

These results imply that, in general, peak expression of XTH family members is at approximately 40 DAA, while the fruit is ripening. These results are consistent with the behavior of the XTH family in tomato, where this family is differentially expressed between mature green and ripe fruits.

2.3. **Gene specificity.** An additional parameter to measure global properties of the transcriptome, apart from diversity and specialization, is the specificity of the genes[16]. Gene specificity, S, is a coefficient which yields a value of 0 for genes which are equally expressed during each of the time points sampled and reaches a maximum value of $\log_2(k)$ where k,

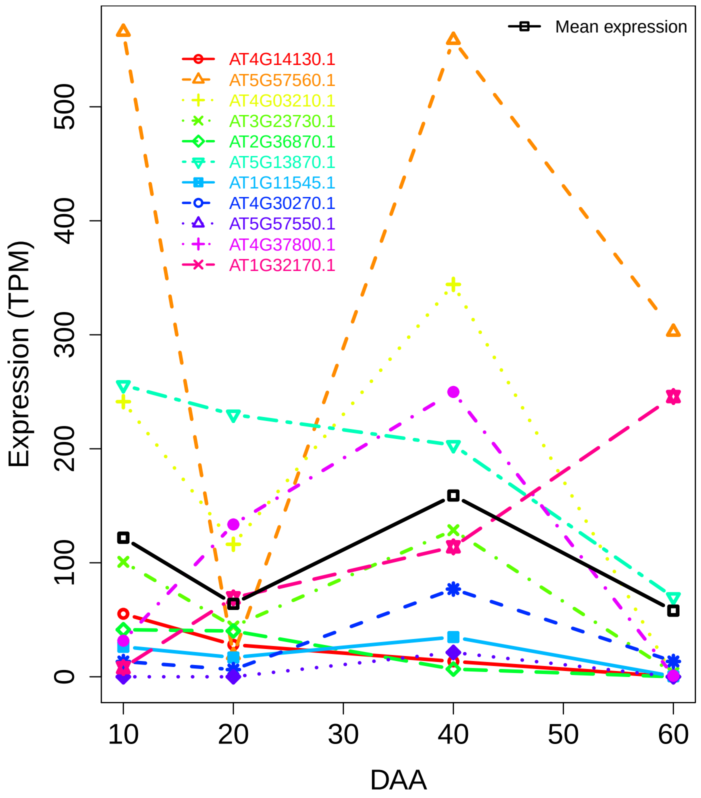

FIGURE AF1-1. Expression of members of the *Xyloglucan endotransglucosylase/hydrolase* family.

is the number of studied transcriptomes for genes expressed exclusively at one state. In our case, the number of time points was $k = 4$, thus the maximum value of S is $\log_2(4) = 2$ for genes expressed in only one of the four stages sampled. Figure AF1-2 presents the distribution of the values of S found in this study.

In Figure AF1-2 the distribution of S is multimodal, with the largest proportion of genes, 8,683 (25%) exhibiting very small specificity values between 0 and 0.1. Peaks were observed at 0.4-0.5 (2,895; 8%), 1.0-1.1 (4,418; 13%) and 1.9-2.0 (5,988; 18%). This complex distribution reflects the structure of the transcriptome during fruit development. A large number of generalist genes are expressed in all four stages (the first class, with 25% of the genes). A second, significant proportion of genes are almost exclusively expressed in one of the stages of development. For example, the 5,988 (18%) genes with specificity near the maximum of 2, are typical members of this group. Interestingly, the group in the middle of the distribution (1.0-1.1) includes a large proportion of genes (13%), that were expressed in only two of the developmental stages. Genes whose expression was detected in three

of the four stages should exhibit a theoretical specificity near 0.5 and, in fact, a peak at 0.4-0.5 corresponds to 8% of the genes.

Combining this information regarding gene specificities, S, with information about the significance of the changes in each one of the three periods of development is a powerful tool to infer the biological roles of the genes.

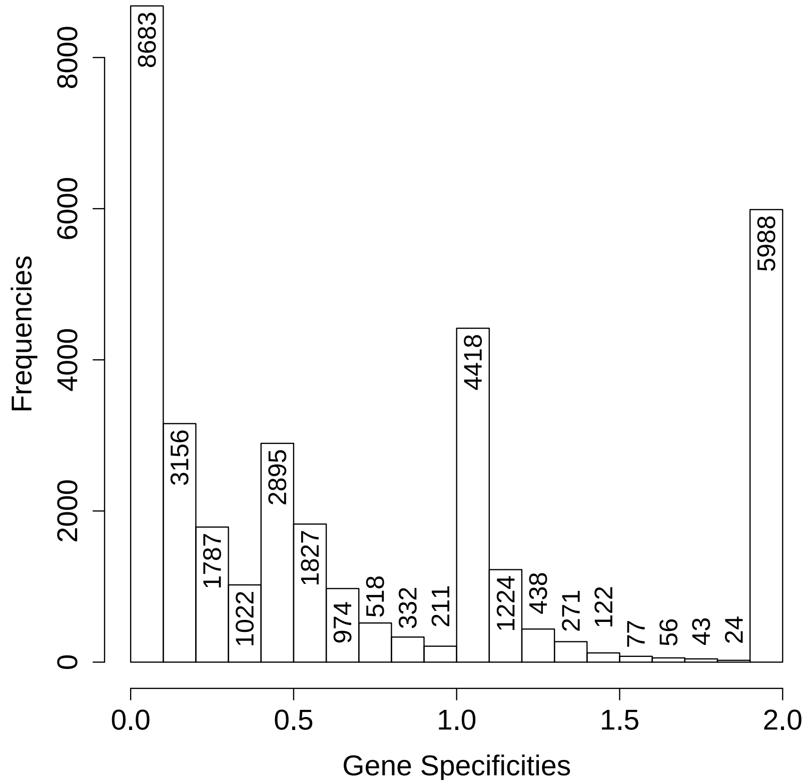

Figure AF1-2. Histogram of gene specificities

2.4. **Genes group by Biological Process (BP) or Metabolic Pathway (MP).** As an example of how this transcriptome data can be mined for useful biological information, we examined the expression of genes grouped into the "Fruit maturation" BP. This BP did not show significant changes in expression when the averaged expression levels of all the genes participating in the group was considered. The patterns of expression exhibited by individual genes are nonetheless interesting.

2.4.1. *Analysis of genes grouped into Fruit maturation BP.* Figure AF1-3 presents the expression of genes classified within the *Fruit maturation* BP as well as the averaged expression of genes participating in this BP. The averaged expression of genes grouped

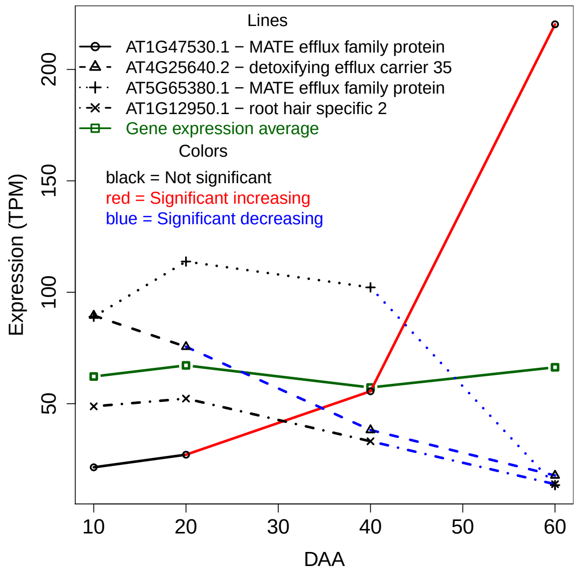

Figure AF1-3. Expression of genes related with Fruit maturation (BP). Chili pepper genes were annotated according to their high degree of similarity to Arabidopsis orthologs. Significance of the expression change is indicated by line color (except for the non-significant change of the average expression per interval, shown in green).

into the Fruit maturation category presented only a very small variation over time, which explains why this BP was classified as "non-significant" at 0.01 FDR. However, each of the four genes that participate in this group exhibited a significant change in expression during at least one interval. In the first interval, between 10 and 20 DAA, all genes maintained a steady-state level of expression. In the second interval, between 20 and 40 DAA, orthologs of two genes, AT5G65380 and AT1G12950 stayed in a relatively steady (not-significant) state. However the expression of genes orthologous to AT1G47530 and AT4G25640 increased or decreased, respectively. In the third interval, from 40 to 60 DAA, all four genes exhibited a significant change in expression. The most influential gene of the four in determining the averaged expression of the group, i.e. the one with the highest absolute expression level, was orthologus to AT1G47530 and encodes a MATE efflux family protein. These proteins have functions in ripening, drug transmembrane transporter or antiporter activities and are localized to the plasma membrane. The other three genes are orthologous to AT4G25640, AT5G65380 and AT1G12950 and were annotated as a detoxifying efflux carrier 35, other member of the MATE efflux family protein and "root hair specific 2", respectively. Interestingly, the gene AT1G12950 (RSH2) had two different loci matches corresponding to two gene models, the first, "root hair specific 2" and the second as a MATE efflux family protein, as two of the other genes; evidently this annotation is the most likely in this case. AT4G25640, annotated as detoxifying efflux carrier 35, was

reported to be involved in flavonoid metabolism and seed development and germination, but has also an annotatation as a MATE efflux family protein. In conclusion, some of the genes involved in fruit maturation in chili pepper are members of the MATE efflux family protein and present significant changes of expression mainly in the third interval, from 40 to 60 DAA.

The previous example shows how it is useful to examine the expression of genes individually as well as within groups of related functions in order to gain a better understanding of the underlying biological process.

2.4.2. *Analysis of genes grouped into "Developmental growth" BP.* As another example of data mining the transcriptome data, Figure AF1-4 shows the expression of genes annotated in the "Developmental growth" BP category. The pattern of change of this BP was significant during all three intervals, increasing in the first and decreasing in the following two intervals (green dashed line in Figure AF1-4). 32 genes with diverse patterns of expression were grouped into this BP, and their levels of expression are shown in Figure AF1-4. However, the pattern of expression of the BP was dominated by a gene orthologous to AT4G21960 from Arabidopsis (upper orange line in Figure AF1-4). This gene had an estimated expression of 3,236, 4,477, 748 and 2 TPM at the four time points sampled (10, 20, 40 and 60 DAA, respectively). Thus this gene was very abundantly expressed at the first two time points but was almost completely shut off at 60 DAA. AT4G21960 encodes a protein from the peroxidase superfamily involved in cellular cation homeostasis and water transport and is located in the extracellular region.

2.4.3. *Analysis of genes grouped into "Capsaicinoids biosynthesis" MP.* The pattern resulting from the summed expression of the 13 genes related to Capsaicinoid biosynthesis showed a significant increase from 10 to 20 DAA, followed by a significant decrease in the interval between 20 to 40 and culminated in a non-significant steady state between 40 to 60 days (pattern $20 - IDS$). This pattern of expression was shared with genes grouped in the "Glycolysis / Gluconeogenesis" MP. All genes grouped into the capsaicinoid biosynthesis pathway were curated using known *Capsicum* genes from distinct databases (see Methods).

Table AF1-6 presents the pattern of expression and description for the genes grouped into the Capsaicinoid biosynthesis MP, while Figure AF1-5 shows the expression in TPM for the 13 genes grouped into this pathway as well as the mean expression for all genes.

From the averaged expression level of genes involved in Capsaicinoid biosynthesis (black line in Figure AF1-5, B) as well as from the estimated expression level of each gene presented in Table AF1-6, the most abundant expression was observed at 20 DAA (pattern $20 - IDS$). This pattern was highly influenced by the behavior of the gene encoding an Acetyl-Coenzyme A Synthetase (ACS), which significantly increased during development, from 220 to 1,838 TPM (row 4 in AF1-6). ACS are reported to participate in fatty acid and glycerolipid metabolism in Arabidopsis [21]. However, the gene which was the key for

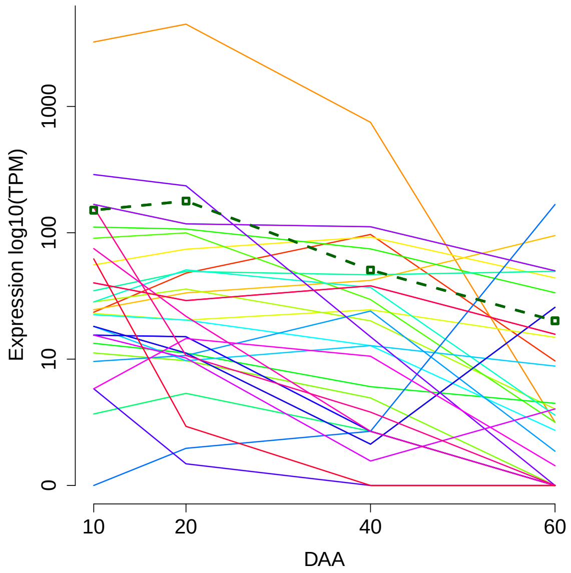

FIGURE AF1-4. Expression of genes related to Developmental growth. Colors denote distinct genes. Change of the average expression per interval is shown as the dashed green line.

capsaicinoid biosynthesis, Pun1, which encodes a putative acyltransferase [22] (row 13 in AF1-6), presented with the pattern $19 - IDD$ (see row 13 of Table AF1-6 and Figure AF1-5, A). Expression of this gene was not detected at 10 DAA, then reached a maximum of 219 TPM at 20 DAA. Expression further decreased to 14 TPM at 40 DAA and was undetectable by 60 DAA. From this we can infer that capsaicinoid biosynthesis is at its highest level around 20 DAA.

2.4.4. *Analysis of genes related to "Ascorbic acid biosynthesis".* Seven genes were grouped into the Ascorbic acid biosynthesis metabolic pathway, and this group as a whole presented a pattern of expression with a significant increase in the first period, between 10 and 20 DAA, and significant decreases in the intervals between 20 to 40 and 40 to 60 DAA ($19 - IDD$). This pattern of expression was shared with three additional MPs, namely Circadian rhythm, Cutin, suberine and wax biosynthesis and Polycyclic aromatic hydrocarbon degradation. Incidentally, the MP "Ascorbate and aldarate metabolism", grouping 13 genes, did not show significant changes of expression as a group during the intervals sampled. It is interesting to note that, as in the case of Capsaicinoid biosynthesis, the maximum level of expression of genes related to Ascorbic acid biosynthesis was observed at 20 DAA. Table AF1-7 and Figure AF1-6 present a description and expression levels of the seven genes involved in Ascorbic acid biosynthesis.

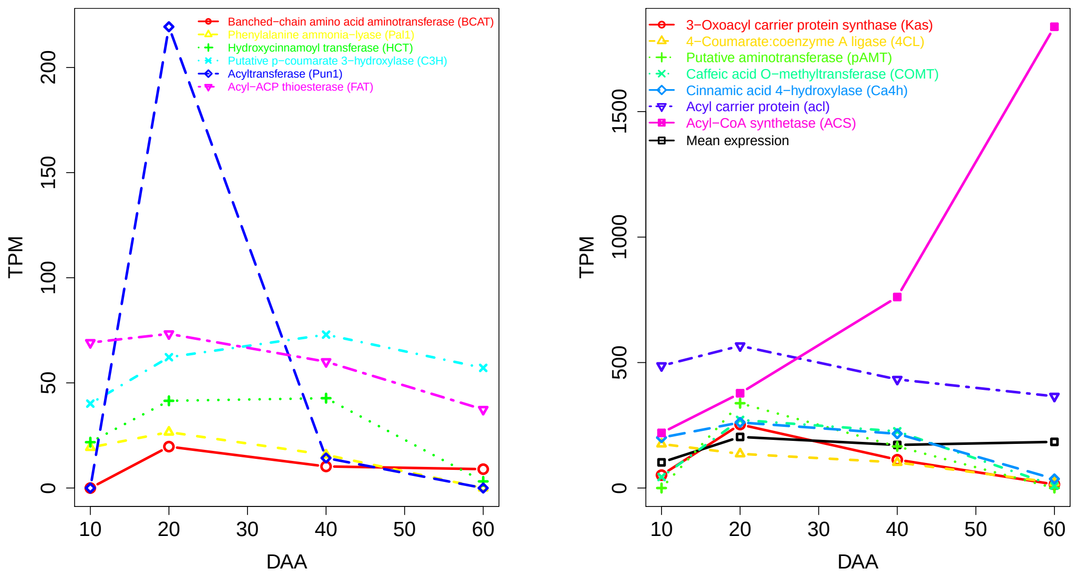

(A) (B)

Figure AF1-5. Expression of genes related to Capsaicinoid Biosynthesis (BP). Mean expression of all genes represented by black line.

Table AF1-6. Estimated expression of genes related with Capsaicinoid biosynthesis (TPM). Average ($\bar{X}$) and Standard deviation (S) for two biological replicates in each case.

Row	Orthologous Gene	10 DAA		20 DAA		40 DAA		60 DAA	
		$\bar{X}$	S	$\bar{X}$	S	$\bar{X}$	S	$\bar{X}$	S
1	Caffeic acid O-methyltransferase (COMT)	44	8	272	5	226	11	9	5
2	4-Coumarate:coenzyme A ligase (4CL)	176	9	136	16	102	1	21	3
3	Acyl carrier protein (acl)	487	14	567	5	433	65	366	33
4	Acyl-CoA synthetase (ACS)	220	15	378	7	761	28	1,838	31
5	Branched-chain amino acid aminotransferase (BCAT)	0	0	20	4	10	1	9	2
6	Putative p-coumarate 3-hydroxylase (C3H)	40	2	62	16	73	13	57	6
7	Cinnamic acid 4-hydroxylase (Ca4h)	201	13	261	12	216	21	36	2
8	Acyl-ACP thioesterase (FAT)	69	8	73	16	60	8	37	6
9	Hydroxycinnamoyl transferase (HCT)	22	3	41	11	43	5	3	2
10	3-Oxoacyl carrier protein synthase (Kas)	52	17	253	12	113	6	14	4
11	Phenylalanine ammonia-lyase (Pal1)	19	15	27	13	16	21	0	0
12	Putative aminotransferase (pAMT)	0	0	338	25	166	14	0	0
13	Acyltransferase (Pun1)	0	0	219	6	14	6	0	0
Average		102	2	204	1	172	1	184	4

The most prevalent pattern of change in the genes involved in Ascorbic acid biosynthesis was to stay steady during the first two periods sampled, from 10 to 40 DAA, and decrease significantly in the last period, from 40 to 60 DAA. Two of the genes (rows 2 and 4 in Table AF1-7) do not present significant changes in expression, while the most variable pattern of expression was exhibited by GGP (row 6 in Table AF1-7) whose expression increased significantly during 10 to 20 DAA and then decreased during the two following intervals, from 20 to 40 and 40 to 60 DAA. This gene was most influential in driving the averaged expression pattern of the ascorbic acid biosynthesis group as a whole $(19 - IDD)$. In summary, the expression of genes involved in ascorbic acid biosynthesis peaked at 20 DAA and then decreased to reach their minimum level at 60 DAA.

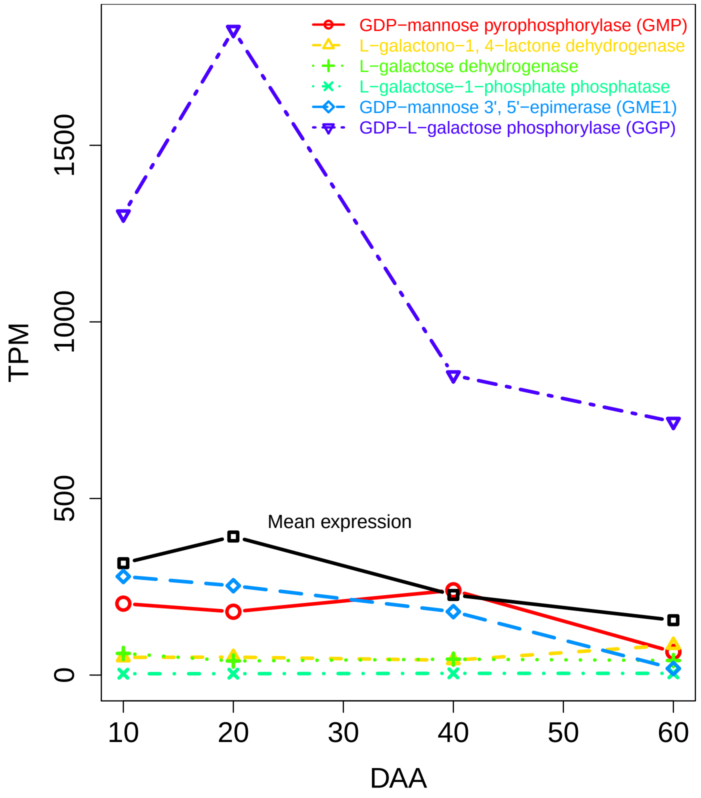

Figure AF1-6. Expression of genes related to Ascorbic Acid Biosynthesis. Mean expression of the genes is indicated as a black line with square symbols

2.4.5. *Expression of genes related to Carotenoid biosynthesis (MP).* Table AF1-8 presents the mean and standard deviation for the expression 9 genes related to carotenoid biosynthesis in TPM, while Figure AF1-7 shows the estimated expression of these genes at the sampled time points.

Table AF1-7. Estimated expression of genes related to Ascorbic Acid Biosynthesis (TPM). Average ($\bar{X}$) and Standard deviation (S) for two biological replicates in each case.

Row	Orthologous Gene	10 DAA		20 DAA		40 DAA		60 DAA	
		$\bar{X}$	S	$\bar{X}$	S	$\bar{X}$	S	$\bar{X}$	S
1	GDP-mannose pyrophosphorylase (GMP)	202	12	179	9	240	13	66	19
2	L-galactono-1, 4-lactone dehydrogenase	50	10	51	6	42	4	85	8
3	L-galactose dehydrogenase	61	4	40	2	45	3	41	0
4	L-galactose-1-phosphate phosphatase	4	1	4	1	5	7	5	2
5	GDP-mannose 3', 5'-epimerase (GME1)	279	1	253	10	180	16	19	1
6	GDP-L-galactose phosphorylase (GGP)	1305	144	1827	98	849	111	717	48
Average		317	19	392	15	227	20	155	13

Table AF1-8. Estimated expression of genes related to Carotenoid Biosynthesis (TPM). Average ($\bar{X}$) and Standard deviation (S) for two biological replicates in each case.

Row	Orthologous Gene	10 DAA		20 DAA		40 DAA		60 DAA	
		$\bar{X}$	S	$\bar{X}$	S	$\bar{X}$	S	$\bar{X}$	S
1	Zeta-carotene/neurosporene desaturase (zds)	85	14	80	3	61	16	124	6
2	Phytoene desaturase (pds1)	107	37	74	9	69	2	133	1
3	Capsanthin/capsorubin synthase	6	6	8	3	8	1	15,206	569
4	Lycopene β-cyclase	20	3	8	2	28	2	69	12
5	Nine-cis-epoxycarotenoid dioxygenase 3	1	1	9	4	9	3	10	3
6	β-carotene hydroxylase	42	8	14	4	16	1	1,709	113
7	Phytoene synthase	10	1	9	1	18	3	157	5
8	Isopentenyl pyrophosphate isomerase	339	73	246	8	247	19	184	3
9	Geranylgeranyl pyrophosphate synthase 1	125	43	31	4	38	2	164	2
Average		82	20	53	0	55	2	1,973	76

The additive expression of genes related to Carotenoid biosynthesis followed the pattern $6{-}DSI$[1]. The fold-change of the average expression from 10 DAA to 60 DAA was estimated as $1,973/82 = 24$, confirming that more abundant expression of genes related to carotenoid biosynthesis is found in the last period of fruit maturation, from 40 to 60 DAA, correlating with the change of fruit color from green to red. The large increase in the average expression during the last period was most influenced by two genes, encoding capsanthin/capsorubin synthase (row 3 in Table AF1-8) and β-carotene hydroxylase (row 6 in Table AF1-8. From Figure AF1-7 panel B, five genes also exhibit a significant increase in expression during

[1]Note that to examine the additive expression is equivalent to examine the *average* expression of the genes, given that only the multiplication by a constant is involved. Plots present the average expression for this to be visible in the same graph than the expression of individual genes.

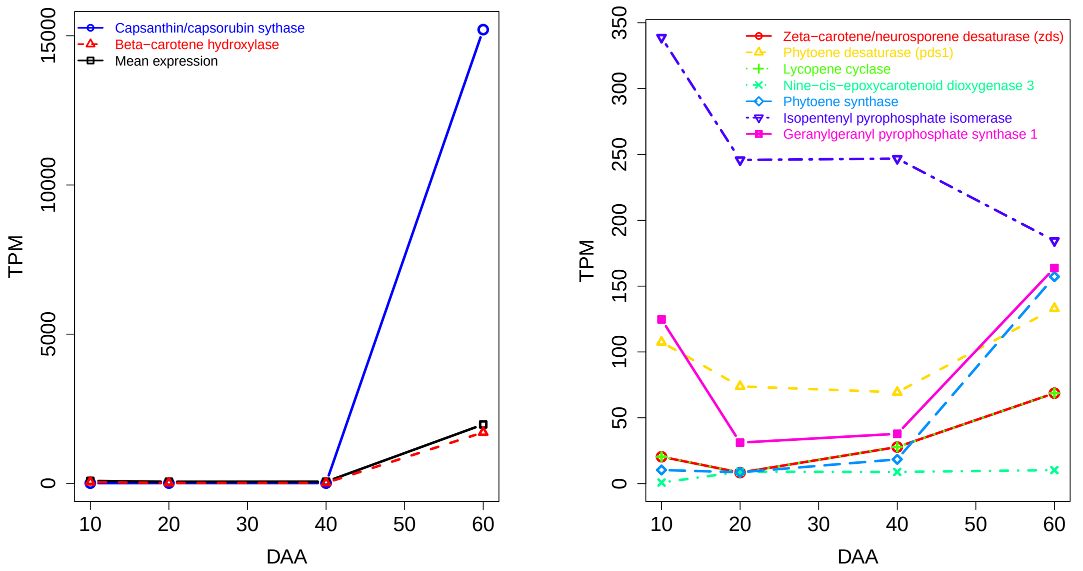

(A) (B)

FIGURE AF1-7. Expression of genes related to Carotenoid Biosynthesis (MP). Mean expression of all genes represented by black line.

the last period of fruit development, from 40 to 60 DAA. Only a single gene, encoding isopentenyl pyrophosphate isomerase (row 8 in Table AF1-8) showed a significant decrease in expression during this period. The pattern of expression for all nine genes related to carotenoid biosynthesis presented in Table AF1-8 was validated by qRT-PCR and these results are presented in the main text.

3. Statistical Analyses: Correction factors for RNA composition

As noted in [20], RNA-Seq gene expression estimates must be normalized to take into account the composition of the sampled RNA populations. If the relative frequencies of expression for a gene are directly employed to estimate fold-changes in expression of the gene, this estimation can be seriously biased [20]. Here we employed a correction, based on the work of Good[8], that potentially removes this bias. The estimated correction factor for removing the bias when comparing libraries "a" *versus* "b", say, $\hat{h}_{ab}$, is given by

$$\hat{h}_{ab} = \frac{\hat{M}_a}{\hat{M}_b}$$

where $\hat{M}_a$ and $\hat{M}_b$ are the estimates of the scaled number of mRNA molecules that conform to the corresponding population (see Table 1 in the main text for the values of $\hat{M}$ in each condition; i.e., at 10, 20, 40 and 60 DAA).

Table AF1-9 presents values for the correction factors used in the statistical test when contrasting two intervals (conditions). Here we employed only the values presented in the main diagonal of Table AF1-9, because we performed comparisons only for neighboring intervals, say 10 *versus* 20, 20 *versus* 40 and 40 *versus* 60 DAA.

TABLE AF1-9. Estimated values of coefficients $\hat{h}_{ab}$.

Condition (DAA)	20	40	60
10	0.903048	1.046679	0.808985
20	–	1.159052	0.895839
40	–	–	0.772907

References

[1] Stephen F Altschul, Thomas L Madden, Alejandro A Schäffer, Jinghui Zhang, Zheng Zhang, Webb Miller, and David J Lipman. Gapped blast and psi-blast: a new generation of protein database search programs. *Nucleic acids research*, 25(17):3389–3402, 1997.

[2] Ana Conesa, Stefan Götz, Juan Miguel García-Gómez, Javier Terol, Manuel Talón, and Montserrat Robles. Blast2go: a universal tool for annotation, visualization and analysis in functional genomics research. *Bioinformatics*, 21(18):3674–3676, 2005.

[3] DIAG. Data intensive academic grid (diag). `http://diagcomputing.org/`.

[4] Jon Duvick, Ann Fu, Usha Muppirala, Mukul Sabharwal, Matthew D Wilkerson, Carolyn J Lawrence, Carol Lushbough, and Volker Brendel. Plantgdb: a resource for comparative plant genomics. *Nucleic acids research*, 36(suppl 1):D959–D965, 2008.

[5] GeneBank. Genbank. `http://www.ncbi.nlm.nih.gov/genbank/`.

[6] GO. The gene ontology. `http://www.geneontology.org/`.

[7] Elsa Góngora-Castillo, Rubén Fajardo-Jaime, Araceli Fernández-Cortes, Alba E Jofre-Garfias, Edmundo Lozoya-Gloria, Octavio Martínez, Neftalí Ochoa-Alejo, and Rafael Rivera-Bustamante. The capsicum transcriptome db: a hot tool for genomic research. *Bioinformation*, 8(1):43, 2012.

[8] I. J. Good. The population frequencies of species and the estimation of population parameters. *Biometrika*, 40(3):237–264, Dec 1953.

[9] Manfred G Grabherr, Brian J Haas, Moran Yassour, Joshua Z Levin, Dawn A Thompson, Ido Amit, Xian Adiconis, Lin Fan, Raktima Raychowdhury, Qiandong Zeng, et al. Full-length transcriptome assembly from rna-seq data without a reference genome. *Nature biotechnology*, 29(7):644–652, 2011.

[10] Eva Huala, Allan W Dickerman, Margarita Garcia-Hernandez, Danforth Weems, Leonore Reiser, Frank LaFond, David Hanley, Donald Kiphart, Mingzhe Zhuang, Wen Huang, et al. The arabidopsis information resource (tair): a comprehensive database and web-based information retrieval, analysis, and visualization system for a model plant. *Nucleic acids research*, 29(1):102–105, 2001.

[11] ITAG. International tomato genome sequencing project. `http://solgenomics.net/organism/Solanum_lycopersicum/genome`.

[12] KEGG. Kyoto encyclopedia of genes and genomes. `http://www.genome.jp/kegg/`.

[13] Ben Langmead, Cole Trapnell, Mihai Pop, Steven L Salzberg, et al. Ultrafast and memory-efficient alignment of short dna sequences to the human genome. *Genome Biol*, 10(3):R25, 2009.

[14] Bo Li and Colin Dewey. Rsem: accurate transcript quantification from rna-seq data with or without a reference genome. *BMC bioinformatics*, 12(1):323, 2011.

[15] Guillaume Marçais and Carl Kingsford. A fast, lock-free approach for efficient parallel counting of occurrences of k-mers. *Bioinformatics*, 27(6):764–770, 2011.

[16] Octavio Martínez and M. H. Reyes-Valdés. Defining diversity, specialization, and gene specificity in transcriptomes through information theory. *Proceedings of the National Academy of Sciences of the United States of America*, 105(28):9709–9714, 2008.

[17] Eva Miedes and Ester P Lorences. Xyloglucan endotransglucosylase/hydrolases (xths) during tomato fruit growth and ripening. *Journal of plant physiology*, 166(5):489–498, 2009.

[18] Yuki Moriya, Masumi Itoh, Shujiro Okuda, Akiyasu C Yoshizawa, and Minoru Kanehisa. Kaas: an automatic genome annotation and pathway reconstruction server. *Nucleic acids research*, 35(suppl 2):W182–W185, 2007.

[19] Kim D Pruitt, Tatiana Tatusova, and Donna R Maglott. Ncbi reference sequences (refseq): a curated non-redundant sequence database of genomes, transcripts and proteins. *Nucleic acids research*, 35(suppl 1):D61–D65, 2007.

[20] Mark D Robinson and Alicia Oshlack. A scaling normalization method for differential expression analysis of RNA-seq data. *Genome biology*, 11(3):R25, January 2010.

[21] Jay M Shockey, Martin S Fulda, et al. Arabidopsis contains nine long-chain acyl-coenzyme a synthetase genes that participate in fatty acid and glycerolipid metabolism. *Plant physiology*, 129(4):1710–1722, 2002.

[22] Charles Stewart, Byoung-Cheorl Kang, Kede Liu, Michael Mazourek, Shanna L Moore, Eun Young Yoo, Byung-Dong Kim, Ilan Paran, and Molly M Jahn. The pun1 gene for pungency in pepper encodes a putative acyltransferase. *The Plant Journal*, 42(5):675–688, 2005.

[23] The Tomato Genome Consortium. The tomato genome sequence provides insights into fleshy fruit evolution. *Nature*, 485(7400):635–641, May 2012.
