# Supplementary material for: Dynamics of the chili pepper transcriptome during fruit development
Source: BMC Genomics. 2014 Feb 21;15:143. doi: 10.1186/1471-2164-15-143 (PMC3936869; doi:10.1186/1471-2164-15-143)
Supplement: Additional file 5 — Gene-specific primers for the qRT-PCR analysis. Primers designed for the qRT-PCR analysis of 9 genes related to carotenoid biosynthesis. [file 1471-2164-15-143-S5.pdf]

Unigene ID	Putative Function	Orientation	Start	Primer Length	Tm	GC%	Sequences 5'-3'	Product Size
**comp30344_c0_seq1**	Geranyl-diphosphate synthase	FORWARD	482	20	60	60	CGTAGCTTCTCCACCTCGAC	85
**comp30344_c0_seq2**		REVERSE	556	20	60	50	ACAAAACGGCAGCTCTCCTA	
**comp27538_c0_seq1**	Beta-carotene hydrolase	FORWARD	753	20	60.2	55	GCATATGCACGAGTCACACC	129
**comp27538_c0_seq2**		REVERSE	881	20	60.2	50	GGGATGAGGCCTTTATGGTT	
**comp9344_c0_seq1**	Capsanthin/capsorubin synthase	FORWARD	600	20	60.1	50	TCTATCCGAAGGCCACAAAC	200
**comp9344_c0_seq1**		REVERSE	799	18	60.2		CGCGGATTCCTCAAAATG	
**comp25714_c0_seq2**	Zeta-carotene dehydrogenase	FORWARD	1388	20	60.1	50	ATGTATGTTAGCGGGCTTGC	128
**comp25714_c0_seq2**		REVERSE	1515	20	60	50	TCCAATTCCCTCCACTTCTG	
**comp32924_c0_seq1**	Phytoene desaturase	FORWARD	234	20	60.2		TGATTATCCAAGGCCAGAGC	139
**comp32924_c0_seq2**		REVERSE	372	20	59.9		AGACAAACCACCCAAACCTG	
**comp22555_c0_seq1**	Phytoene synthase	FORWARD	41	20	60	50	TCTCAAACGGGACAGGATTC	122
**comp22555_c0_seq1**		REVERSE	162	20	59.4	50	CCACCTTTGTTTTCCACCTC	
**comp18026_c0_seq2**	Zeaxanthin epoxidase	FORWARD	1249	20	60.4	50	TATTTGGGGGATGGTGTGAC	163
**comp18026_c0_seq2**		REVERSE	1411	20	60	50	CCCAAATTAGGCTGCATAGC	
**comp19500_c0_seq1**	Isopentenyl pyrophosphate isomerase	FORWARD	469	20	59.6	50	CTTCAGCAACGATCAGCAAC	71
**comp19500_c0_seq2**		REVERSE	539	19	59.8	52.6	AGTGGATGGCTGCAACAAG	
**comp26908_c1_seq5**	Lycopene cyclase	FORWARD	1184	20	60.3	50	AGTTATCCGCAGCTGTTTGG	160
**comp26908_c1_seq5**		REVERSE	1343	20	60.3	50	CCATGCCAATAACGAGGTTC	
